# ICU readmission and mortality risk prediction: Generalizability of a multi-hospital model

**DOI:** 10.1016/j.jointm.2025.03.007

**Published:** 2025-06-14

**Authors:** Tariq A. Dam, Daan de Bruin, Giovanni Cinà, Patrick J. Thoral, Paul W.G. Elbers, Corstiaan A. den Uil, Reinier F. Crane

**Affiliations:** 1 Department of Intensive Care, Amsterdam University Medical Center, Amsterdam, The Netherlands; 2 Quantitative Data Analytics Group, Vrije Universiteit, Amsterdam, The Netherlands; 3 Pacmed, Amsterdam, The Netherlands; 4 Department of Medical Informatics, Amsterdam University Medical Center, Amsterdam, The Netherlands; 5 Institute for Logic, Language and Computation, University of Amsterdam, Amsterdam, The Netherlands; 6 Department of Intensive Care, OLVG, Amsterdam, The Netherlands; 7 Department of Intensive Care, Maasstad Ziekenhuis, Rotterdam, The Netherlands

**Keywords:** Artificial intelligence, Intensive care, Patient readmission, Mortality, Patient discharge

## Abstract

**Background:**

Inadvertent intensive care unit (ICU) readmission is associated with longer length of stay and increased mortality. Conversely, delayed ICU discharge may represent inefficient use of resources. To better inform discharge timing, several hospitals have implemented machine learning models to predict readmission risk following discharge. However, these models are typically created locally and may not generalize well to other hospitals or patient populations. A single multi-hospital-based model might provide more accurate predictions and insight into features that are applicable across diverse clinical settings.

**Methods:**

This study involved a retrospective multi-center cohort from one academic hospital (Amsterdam University Medical Center [AUMC]) and two large teaching hospitals (Maasstad Ziekenhuis [MSZ] and OLVG). Data from the latter two hospitals were combined to create a pooled model, which was tested on the academic hospital dataset. Data relating to all adult ICU patients were included, starting from the implementation of the electronic health record system until the commencement of model development for each hospital. An XGBoost model was trained to predict a composite outcome of readmission or mortality within 7 days and an autoencoder was used as an out-of-distribution (OOD) detector to capture dataset heterogeneity.

**Results:**

In total, 44,837 patients were available for analysis across the three hospitals. The average readmission rates were 7.1 %, 6.9 %, and 5.9 % for MSZ, OLVG, and AUMC, respectively. Performance evaluation of the local models on AUMC data demonstrated weighted area under the receiver operating characteristic curves of 69.7 %±0.8 %, 70.5 %±0.5 %, and 76.5 %±1.9 %, respectively, whereas the pooled model achieved a weighted area under the receiver operating characteristic curves of 71.1 %±0.7 %. The difference between internal and external performance was reduced when cardiac surgery patients were excluded. The key features across models were albumin levels and the use of oxygen therapy.

**Discussion:**

A single, multi-hospital-based model performed comparably on external datasets, especially when cardiac surgery patients were excluded. However, when applied externally, model predictions risk being uncalibrated for specific patient subgroups and require careful calibration before implementation. While external models were more stable than local ones over OOD scores, their performance was comparable after excluding cardiac surgery patients. Although pooling data marginally improved performance on external datasets, the incorporation of data from diverse hospitals is likely to provide greater benefits.

## Introduction

A decline in intensive care unit (ICU) capacity over time puts pressure on the availability of ICU beds, especially given the expected increase in demand.^[^^1^^,^^2^^]^ In addition, ICU care is more expensive than care on regular wards,^[^^3^^]^ and as a society, we face a significant challenge in ensuring that high-quality ICU care remains accessible and affordable.

ICU capacity can be optimized by discharging patients as soon as medically safe.^[^^4^^]^ However, discharging patients too early may lead to higher readmission rates, increased mortality, and longer overall length of stay.^[^^5^^,^^6^^]^

In three hospitals in The Netherlands, insights into ICU readmission risk are already available and integrated into clinical practice as a decision support tool using an explainable machine learning model.^[^[7], [8], [9]^]^ This model, developed under the brand name Pacmed Critical, is implemented locally and uses clinical data, such as vital signs and information from organ support systems, derived from each hospital’s historic and live patient data.^[^^10^^]^

The use of each hospital’s unique dataset is essential, given that machine learning models often exhibit poor external validity.^[^^11^^,^^12^^]^ Although predictive performance can be improved through further training on the specific local population,^[^^13^^]^ implementation is time-consuming and costly. Furthermore, hospitals with a limited amount of data may find it challenging to achieve the desired performance through model retraining. Even with sufficient overall training data, specific subpopulations may be underrepresented, potentially compromising the reliability of predictions.^[^^13^^]^

A potential solution for these challenges is training models on data from multiple hospitals, as combined datasets might better capture heterogeneity, potentially resulting in more generalizable models.^[^^14^^]^ A good performance on an external dataset serves as an indicator, although not a guarantee, of a model’s generalizability.^[^^15^^]^

Our main goals in this study were to evaluate the external validity of Pacmed Critical across different hospitals using area under the receiver operating characteristic curves (AUROC) scores, as well as improve its external validity by training the model on pooled data from multiple hospitals. Heterogeneity across hospitals was compared by using out-of-distribution (OOD) detection methods, while performance was evaluated over OOD scores. In addition, we sought to identify which features contribute to the generalizability of the model across hospitals.

## Methods

Following a review, the local Medical Ethics Committee of Amsterdam University Medical Center (AUMC) and the Bureau of Research and Innovation of the Santeon Working Group determined that the study protocol was outside the scope of the Dutch Medical Research Involving Human Subjects Act (WMO) and waived the need for informed consent. This report adheres to the TRIPOD-AI reporting guidelines.^[^^16^^]^

### Study population

The participating hospitals comprised one University Medical Center with a 32-bed ICU (AUMC, location VUmc) and two large teaching hospitals with 24-bed (OLVG) and 20-bed (Maasstad Ziekenhuis [MSZ]) ICUs. All ICUs were mixed surgical-medical units. All patients admitted to the ICUs of participating hospitals from the inception of their electronic health record systems until data extraction for Pacmed Critical implementation were included in the study. After patient selection, pseudonymization and anonymization were applied to remove identifiable personal information.

### Feature selection

The development of the initial model involved feature selection based on predictive performance, collinearity, and expert opinion, as previously described.^[^^7^^]^ From the resulting set, features were further selected based on their availability and consistent relevance across all hospitals. Some features, such as measurement counts, were excluded, while others, such as the occurrence of cardiac enzyme measurements, were transformed. The final list of included features is available in Supplementary Table S1.

### Data processing

Clinically relevant features, such as the mean, minimum, maximum, and last blood pressure over the previous 24 h, were obtained from routinely collected clinical data in patient records. For the composite outcome measure of readmission or mortality within 7 days, the time until the patient’s next ICU admission or the registered time of death, if available, was considered. The dataset was then split into a training set and a test set with an 80/20 split, ensuring that patients with multiple admissions were placed within the same set. Missing data were imputed using fixed values for features that were not missing at random and median values for all other features (Supplementary Table S1). Data were standardized and scaled by subtracting the mean and scaling to one standard deviation. Categorical data underwent one-hot encoding.

### Modeling

An Extreme Gradient Boosting (XGBoost) classifier was used to predict the composite outcome of ICU readmission or mortality within 7 days of ICU discharge (see also Methods - Endpoints). In total, four prediction models were trained: one for each hospital, plus one that was trained on a pooled dataset combining data from MSZ and OLVG. Each prediction model was evaluated on all datasets (Supplementary Figure S1). Hyperparameter settings were consistent with those applied in the initial product implementation across all datasets (Supplementary Table S2).

Hospital-specific retraining and calibration were previously identified as key steps for applying models in new settings.^[^^13^^]^ However, given that the aim of the present study was to improve external validity and evaluate the need for hospital-specific recalibration by training on data from multiple sources, uncalibrated models were used to evaluate differences in predicted probabilities.

To assess the similarities in patient populations across hospitals and evaluate potential model failure in new settings,^[^^17^^]^ OOD models were trained using an autoencoder for each dataset, using the same set of features (Supplementary Table S3). Each OOD model was trained on a specific hospital dataset and learned to reconstruct patient data. The reconstruction error served as an OOD score, quantifying how different an individual patient was from the training distribution.^[^^18^^]^ A high OOD score indicated that a patient’s features deviated substantially from those of the training cohort, suggestive of a greater likelihood of poor model performance due to data distribution shifts.

One OOD model was trained per hospital, and an additional OOD model was trained using the pooled dataset. These OOD scores were then used to illustrate patient population similarities across datasets and stratify prediction results by OOD score, allowing investigation into whether model performance degraded in patients with higher OOD scores.^[^^19^^,^^20^^]^ This approach provides a practical measure of generalizability and potential failure detection before clinical deployment.

### Endpoints

The primary outcome was external predictive performance for the composite readmission or mortality within 7 days of ICU discharge as assessed by AUROC scores, with stratifications for OOD score and admission specialty. ICU readmission was defined as a transfer from the ICU to the general ward and back during the same hospital stay. Patients in palliative care, patients with do-not-resuscitate or do-not-intubate orders, patients with a length of stay under 12 h or over 30 days, and patients transferred to other hospitals were excluded from the analysis.^[^^7^^]^

The composite outcome of readmission or mortality within 7 days was chosen because the transition of care to a less monitored environment may lead to preventable errors and adverse events,^[^^7^^]^ indicating an overall failure of ICU discharge planning and post-ICU care strategies. Combining these endpoints provides a more comprehensive assessment of discharge safety, especially as discharge criteria may vary across hospitals.^[^^21^^]^

All estimates were obtained using 5-fold cross-validation with different splits of training and testing data and are reported as averages with standard deviation. The random seed for the train/test split was kept consistent across the datasets, ensuring that the pooled model was trained on the same set of data from OLVG and MSZ that was used for the individual hospital models. Given that the proportion of cardiac surgery patients was markedly higher in two of the datasets than in the other, a sensitivity analysis was performed after the exclusion of these patients from all datasets.

To facilitate interpretation, the AUROC score for each prediction model was compared to its AUROC score on the local dataset (the local model), with the difference expressed as the AUROC-delta. Contributions to the predictions were evaluated using feature importance based on SHapley Additive exPlanation (SHAP) values.^[^^22^^]^

## Results

### Population

During product implementation, data were extracted from three hospitals, resulting in the inclusion of 19,417 patients from OLVG (January 2008 to November 2022), 10,092 from MSZ (June 2008 to February 2023), and 15,328 from AUMC (January 2004 to October 2022). The incidence of the primary outcome varied from 5.9 % for AUMC to 7.1 % for MSZ. Based on this incidence and the use of 52 features, the required sample size ranged between 7300 and 8800 patients.^[^^23^^]^

Characteristics for the entire dataset are described in Table 1 and Supplementary Table S4. Patient characteristics stratified per hospital are provided in Supplementary Tables S5 and S6. Regarding ICU specialties, OLVG and AUMC shared a similar distribution of admissions, both showing a high proportion of cardiac surgery patients, followed by surgery and internal medicine patients (Supplementary Table S5). Conversely, MSZ, which does not perform cardiac surgery, had a predominantly medical ICU.^[^^24^^]^ The characteristics of cardiac surgery patients are listed in Supplementary Table S7 and those of the remaining patients in Supplementary Tables S8 and S9.

**Table 1:** Patient characteristics, reduced to admission level data and outcomes.

Parameter	Value	Missing data
Age (years)		0 (0.0)
<50	6744 (15.04)	
50–70	20,591 (45.92)	
>70–80	13,270 (29.60)	
>80	4232 (9.44)	
Sex		289 (0.60)
Female	15,114 (33.71)	
Male	29,434 (65.65)	
BMI (last 24 h, kg/m^2^)	26.25 (23.67–29.41)	18,464 (41.20)
Hospital name		
MSZ	10,092 (22.51)	
OLVG	19,417 (43.31)	
AUMC	15,328 (34.19)	
ICU specialty		
Cardiac surgery	18,765 (41.85)	
Unknown	10,095 (22.51)	
Surgery	4103 (9.15)	
Internal medicine	2954 (6.59)	
Cardiology	2208 (4.92)	
Pulmonary	1970 (4.39)	
Neurology	793 (1.77)	
ICU	646 (1.44)	
Neurosurgery	571 (1.27)	
Gastroenterology	543 (1.21)	
Vascular surgery	477 (1.06)	
Other	1712 (3.82)	
Length of stay		
4–12 h	12 (0.03)	
12–24 h	19,767 (44.09)	
1–3 days	14,555 (32.46)	
3–7 days	5993 (13.37)	
7–14 days	2609 (5.82)	
>14 days	1901 (4.24)	
Outcomes		
Readmission <7 days	2494 (5.56)	
Readmission or mortality <7 days	2967 (6.62)	
Mortality, after ICU discharge		
7 days	629 (1.40)	
30 days	1500 (3.35)	
90 days	2135 (4.76)	

Data are presented as *n* (%) or median (interquartile range).

Full table is available in online Supplementary Table S4. Mortality is calculated from the moment of discharge and within the specified window, regardless of whether the patient was admitted to the ICU or regular ward.

BMI: Body mass index; ICU: Intensive care unit.

### Modeling

Local model performance yielded AUROCs between 70.9 % and 76.5 % (Table 2). Performance was lower for models trained on external datasets. However, the performance of the OLVG model on MSZ data was comparable to the MSZ model, suggesting that the characteristics of MSZ patients are represented within a subset of the OLVG population. The pooled model achieved the same performance as the respective local models when applied to MSZ and OLVG data, and showed the smallest decrease in performance on the AUMC dataset (–5.4 % *vs.* –6.0 % and –6.8 %, respectively), indicating that pooling data may enhance generalizability (Table 2).

**Table 2:** Performance by AUROC score for each model on each testing dataset.

Test dataset	Model	AUROC	AUROC-delta
MSZ	MSZ	70.9±1.4	
	OLVG	70.5±1.7	–0.3±1.2
	Pooled*	70.9±1.6	–0.0±1.0
	AUMC	69.2±1.3	–1.7±1.5
OLVG	OLVG	74.0±1.5	
	MSZ	68.9±2.6	–5.0±2.8
	Pooled*	74.0±1.4	0.1±0.3
	AUMC	71.0±1.8	–3.0±0.5
Pooled*	Pooled*	72.9±1.4	
	MSZ	69.5±1.6	–3.3±1.4
	OLVG	72.3±1.6	–0.5±0.3
	AUMC	69.8±1.5	–3.1±0.2
AUMC	AUMC	76.5±1.9	
	MSZ	69.7±0.8	–6.8±1.7
	OLVG	70.5±0.5	–6.0±2.0
	Pooled*	71.1±0.7	–5.4±2.1

AUROC: Area under the receiver operating characteristic curves; AUMC: Amsterdam University Medical Center; MSZ: Maasstad Ziekenhuis.

Data are presented as weighted means±standard deviation, averaged over 5-folds. AUROC-delta was calculated against the model of the corresponding dataset. Sample sizes and incidence rates are reported in Table 4.

⁎ The pooled dataset consists of the combined data from MSZ and OLVG and the pooled model is trained on the training data of these hospitals. Model performance confidence intervals are reported in Supplementary Table S10.

When stratifying predictions by cardiac surgery (CTC) and non-cardiac surgery (non-CTC), the biggest drops in performance across models were primarily observed in the CTC subgroup, with a smaller drop in performance on non-CTC patients, showing that the generalizability of some patient subgroups across hospitals may be limited. However, within the non-CTC group, the pooled model achieved the best performance on OLVG data, suggesting that augmenting a local dataset with external data may be beneficial (Table 3)

**Table 3:** Model performance by AUROC score on OLVG and AUMC testing datasets, stratified over admission specialty grouped by CTC and non-CTC.

Test dataset	Model	CTC		non-CTC	
		AUROC	AUROC-delta	AUROC	AUROC-delta
OLVG	OLVG	71.6±2.8		69.5±2.3	
	MSZ	64.1±3.1	–7.5±3.5	68.5±3.5	–0.9±1.8
	Pooled*	71.5±2.7	–0.2±0.4	69.9±2.4	0.5±0.6
	AUMC	65.9±3.4	–5.7±1.9	66.4±2.2	–3.1±1.4
AUMC	AUMC	77.3±3.2		68.1±1.3	
	MSZ	70.9±6.6	–6.4±3.8	66.9±0.9	–1.2±0.8
	OLVG	75.6±3.5	–1.8±3.1	66.1±1.3	–2.1±1.2
	Pooled*	75.9±2.9	–1.4±2.2	66.6±1.0	–1.6±0.9

AUROC: Area under the receiver operating characteristic curves; AUMC: Amsterdam University Medical Center; CTC: Cardiac surgery; MSZ: Maasstad Ziekenhuis.

Data are presented as weighted means ±standard deviation, averaged over 5-folds. Results for testing on MSZ were omitted due to the absence of cardiac surgery patients. AUROC-delta was calculated against the model of the corresponding dataset. Sample sizes and incidence rates are reported in Table 5. AUROC scores stratified by admission specialty are reported in Supplementary Table S11. Model performance when cardiac surgery patients from the training and testing datasets were excluded are available in Supplementary Tables S12 and S13.

* The pooled dataset consists of the combined data from MSZ and OLVG and the pooled model was trained on the training data of these hospitals. Model performance confidence intervals are reported in Supplementary Table 10.

Average predicted probabilities were accurate for the local models on their respective datasets but were under- and overestimated when applied to external datasets, indicating a need for calibration (Table 4). When stratified by CTC status, the readmission risk for CTC patients from AUMC was overestimated 3–4-fold by models from the other hospitals, showing that calibration requirements varied across patient groups (Table 5).

**Table 4:** Average predicted probability of the prediction models on each dataset and the dataset’s incidence.

Test Dataset	MSZ Model	OLVG Model	Pooled* Model	AUMC Model	Sample size	Incidence
MSZ	7.0±0.1	9.5±0.4	7.6±0.2	8.0±0.2	1991.8±0.4	7.1±0.0
OLVG	5.8±0.2	6.8±0.0	6.5±0.0	5.6±0.2	3844.2±0.4	6.9±0.0
Pooled*	6.2±0.2	7.7±0.2	6.8±0.1	6.4±0.2	5836.0±0.6	6.9±0.0
AUMC	6.8±0.2	10.0±0.5	8.1±0.4	5.8±0.1	3026.8±0.4	5.9±0.0

Data are presented as weighted mean±standard deviation, averaged over 5-folds.

⁎ The pooled dataset consists of the combined data from MSZ and OLVG and the pooled model is trained on the training data of these hospitals. AUMC: Amsterdam University Medical Center; MSZ: Maasstad Ziekenhuis.

**Table 5:** Predicted probability of the prediction models on each dataset, stratified over CTC or non-CTC patients.

Test Dataset	MSZ Model	OLVG Model	Pooled* Model	AUMC Model	Sample size	Incidence
OLVG | CTC	5.2±0.3	4.7±0.1	4.6±0.1	3.7±0.1	2302.6±21.4	4.3±0.2
AUMC | CTC	6.0±0.3	8.5±0.7	6.8±0.6	3.2±0.1	1436.0±18.1	2.2±0.3
OLVG | non-CTC	6.7±0.1	9.9±0.2	9.2±0.1	8.4±0.3	1542.1±21.6	10.8±0.4
AUMC | non-CTC	7.6±0.3	11.3±0.5	9.3±0.3	8.1±0.1	1591.2±18.3	9.2±0.2

Data are presented as weighted mean±standard deviation, averaged over 5-folds.

⁎ The pooled model is trained on the training data of MSZ and OLVG combined. AUMC: Amsterdam University Medical Center; CTC: Cardiac surgery; MSZ: Maasstad Ziekenhuis.

### Data similarity and OOD collinearity

Data similarity, measured as the collinearity between OOD models on a given dataset, ranged from 0.436 to 0.891. The lowest collinearity was observed between the OLVG and MSZ OOD models when evaluated on the pooled dataset, and the highest was noted between the MSZ and AUMC OOD models when assessed on OLVG data (Supplementary Table S14). The lower collinearity observed between the OLVG and MSZ OOD models with respect to their pooled dataset indicates that these hospitals may benefit from pooling data, as each can contribute new data to the prediction model, a benefit reflected in the AUROC-delta (Table 2). The higher collinearity found between the MSZ and AUMC OOD models on OLVG data suggests that there is substantial agreement between these hospitals in their evaluation of OLVG patients. When evaluated on OLVG data, the MSZ and OLVG OOD models showed a lower collinearity (0.481) than the AUMC and OLVG OOD models (0.646), corresponding to their respective AUROC-delta values for the OLVG dataset (Table 2). When cardiac surgery patients were excluded, these correlations changed to 0.874 and 0.853, respectively, indicating that cardiac surgery patients contribute greatly to the data dissimilarity between MSZ and OLVG, which corresponds to changes in predictive performance (Supplementary Table S12).

**Figure 1:**
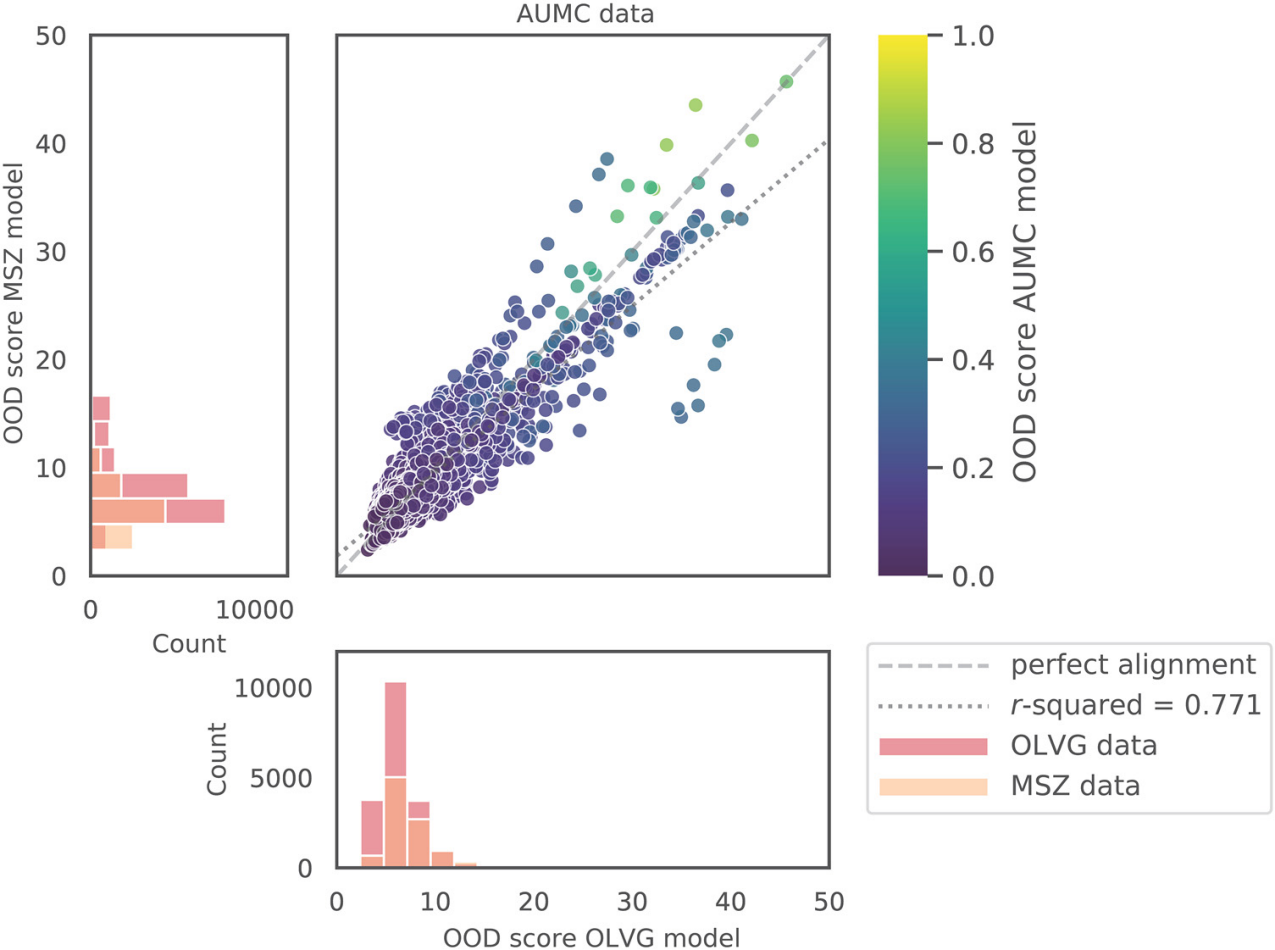
Collinearity of OOD scores and hospital data distribution. The dashed line denotes the perfect alignment of OOD scores on the AUMC dataset and *r*-squared represents the slope of the best-fitted linear model. The histograms show the distribution of OOD scores for each training hospital. Count represents the number of patients within the OOD score bin. AUMC: Amsterdam University Medical Center; MSZ: Maasstad Ziekenhuis; OOD: Out-of-distribution.

With respect to AUMC data, the correlation between the MSZ and OLVG OOD models was 0.771, increasing to 0.867 with the exclusion of cardiac surgery patients, indicating that heterogeneity may be low and that the pooling of data may have a limited effect (Figure 1, Supplementary Tables S14 and S15).

### Model performance by OOD score

Stratified by the OOD scores of the pooled model, the AUMC model showed decreasing performance with increasing OOD scores, while the pooled and individual models maintained a relatively stable, albeit lower, level of performance. Furthermore, the performance of the pooled model appeared to remain close to that of the best-performing individual contributing local model, indicating a potential benefit of data pooling (Supplementary Figure S2-S4, Supplementary Table S16). When CTC patients were excluded, all models achieved comparable scores (Supplementary Figure S5-S7). For patients with the highest OOD scores, that is, those scoring above the 99th percentile, all models exhibited a decline in performance, with no significant difference in the magnitude of the drop between them (Supplementary Tables S17 and S18).

In a clinical setting, this information could be valuable for decision support. For example, if a predictive model is deployed in a hospital with a markedly different patient case mix, an elevated OOD score for a given patient could serve as an alert to clinicians that the model’s prediction should be interpreted with caution, or even disregard the model’s predictions. This would allow clinicians to supplement the model’s output with additional clinical judgment, reducing the risk of over-reliance on potentially biased predictions.

### Predictor importance

The most influential features across all external models were albumin levels, age, urine production, and oxygen therapy. Feature importance showed slight variation between local and pooled models. However, for AUMC, the most salient feature was the presence of a creatine kinase-MB measurement, followed by heart rate, respiratory rate, and albumin levels (Supplementary Figures S8–S11). When cardiac surgery patients were excluded, the key features for AUMC shifted to albumin, heart rate, and oxygen therapy (Supplementary Figure 12).

## Discussion

In this study, by combining datasets from multiple hospitals, we sought to improve the external validity of a clinically implemented machine learning model designed for the prediction of ICU readmission risk.

The OLVG and AUMC prediction models showed similar performance on MSZ data, while a decline in performance was seen in models originating from external hospitals on both OLVG and AUMC datasets. Pooling data from MSZ and OLVG resulted in similar performance on the datasets of the contributing hospitals and led to a marginal increase in performance on the external dataset compared to models trained on data from only one of the contributing hospitals (Table 2).

Following stratification by CTC and non-CTC subgroups, individual hospital models performed similarly on the non-CTC datasets from both OLVG and AUMC, indicating generalizability without pooling data for this subset (Table 3). For the CTC subgroup, the performance of the OLVG model on AUMC data remained comparable to that on its own data, even though the overall performance was lower (Table 2, Table 3).

This decline in overall performance is attributable to the OLVG model overestimating the composite readmission/mortality probability for all AUMC patients, especially for the CTC subgroup (Table 4, Table 5). The pooled model was similarly affected, showing that, even though the overall performance may be comparable, predictions for subgroups of patients may be over- or underestimated. This may indicate data drift, where the relationship between a patient’s condition and their probability of readmission/mortality varies across hospitals, which may be difficult to rectify without additional data.

This may be illustrated through model performance on the CTC subgroups. The model originating from MSZ, which has no cardiac surgery,^[^^24^^,^^25^^]^ showed a drop in performance when evaluated on these patients, while the performance of the OLVG model remained comparable between these subgroups. However, the performance of the AUMC model on OLVG data also declined, even though both hospitals have a similar proportion (circa 50 %) of cardiac surgery patients (Table 3, Supplementary Table S5). This could potentially be explained if AUMC’s CTC dataset represents a subset of OLVG’s. Domain knowledge suggests that a major difference lies in surgical techniques, as OLVG uses predominantly minimally invasive surgery. Furthermore, CTC patients undergo a strictly locally protocolized ICU admission and subsequent discharge process and have low readmission rates, which may explain the performance gap. Furthermore, studies have demonstrated the influence of organizational factors on ICU discharge decisions,^[^^21^^]^ which may explain some of the variation in ICU readmission rates.

The marginal increase in performance observed from pooling data for all patients may partially be explained by limited heterogeneity across hospitals. This low heterogeneity was illustrated through the use of OOD scores to measure the degree of agreement between hospitals on whether patients fall within the distribution of their respective datasets. Hospitals with similar patient populations will show a high collinearity, as shown in Figure 1. This pattern is also observed between local hospitals and corresponds to their predictive performance, with a low collinearity corresponding to a lower predictive performance of individual models and a higher performance of the pooled model. The removal of the CTC group increased both collinearity and predictive performance for MSZ with both OLVG and AUMC (Supplementary Table S14, S15, Table 2, Supplementary Table S12, Table 3, Supplementary Table S13).

This is not the first attempt to improve the external validity of this readmission risk model. An earlier attempt using a single center for initial development showed a moderate discrimination of 72 %, reaching 79 % after retraining on the local dataset,^[^^13^^]^ and seems consistent with our single-center evaluations on external hospitals. In addition, our current approach focused on improving scores without training on the external dataset.

In the current study, we identified albumin levels as being the most consistent top predictor of ICU readmission and mortality across hospitals, except for local model performance in the presence of cardiac surgery patients. Albumin’s association with ICU readmission and mortality is well-documented, although its utility in clinical decision-making is still debated.^[^^26^^]^ Hypoalbuminemia reflects illness severity and has been linked to ICU readmission and mortality, particularly in sepsis.^[^^27^^]^ However, its predictive value varies, and it should be regarded as an associative feature in our model rather than a causal determinant of readmission.

This study has several limitations. Model training was limited to data from two hospitals, and only one hospital was used for testing, which may not be enough to properly unlearn spurious associations in the dataset.^[^^28^^]^ Although the datasets originated from three large hospitals in two major cities in The Netherlands, covering a wide variety of patients nationwide, the results have to be interpreted with caution, as data drift across hospitals remains a possibility. Furthermore, not all features were available across all datasets, and only those available in all datasets were included in model development. Nevertheless, the impact of this constraint was minimal, as the current approach reached an AUROC of 76.4 %, closely matching the AUROC of 78 % for the AUMC model when evaluated on its own dataset.^[^^7^^]^

Although studies have addressed several pitfalls for medical data science and generalizability,^[^^14^^,^^29^^]^ our approach tackles some additional practical challenges across medical datasets. Importantly, data recording frequencies varied across hospitals, not necessarily based on illness severity, but most likely due to local practice differences, requiring a customized imputation strategy. While two hospitals registered creatine kinase-MB levels, they differed in whether they registered mass or activity, and the presence of either was used instead of the measured value. Furthermore, autoencoders, as used in ODD detection, are sensitive to the differences mentioned before, as well as to the skewed distribution of laboratory values, which were log-transformed.

Future research should focus on organizational differences within patient subgroups and across hospitals that were not captured by the electronic health record systems, as well as the acquisition of additional datasets while managing variations in feature availability. Ultimately, prospective cohort studies are needed to evaluate the actual clinical efficacy of these models.

## Conclusion

Single-hospital models of Pacmed Critical show comparable performance on external datasets, especially when cardiac surgery patients are excluded. However, when applied externally, model predictions risk being poorly calibrated over patient subgroups, and require careful calibration before implementation. While external models were more stable than local ones over OOD scores, the difference between them diminished once cardiac surgery patients had been excluded. Although the pooling of data from hospitals resulted in a marginally improved performance on external datasets, the benefits of this approach are likely to be more substantial with the inclusion of data from a wider range of hospitals.

## Acknowledgments

Santeon Working Group: Ilse van Stijn, Sjoerd Niehof, William Boender, Rutger van Meeuwen

Pacmed B.V.: Marijn Kroes, Taco Houwert, Olivier Thijssens, Marco van der Linden, Mattia Fornasa, and all other employees involved in data collection, extraction, and validation.

## Funding

Pacmed B.V. and Santeon Working Group received funding from healthcare insurers CZ and Zilveren Kruis for their collaboration to develop scalable and data-driven technology.

## Ethics Statement

After review, the local Medical Ethics Committee of Amsterdam UMC and the Bureau of Research and Innovation of the Santeon Working Group reviewed the study protocol and determined that the study protocol fell outside the scope of the Dutch Medical Research Involving Human Subjects Act (WMO), and waived the need for informed consent. The Medical Ethics Committee of Amsterdam UMC, location VUmc reviewed the project as project number 2017.212, date: May 2017, study title: “Right Data, Right Now: Predicting ICU readmission rates”. The Bureau of Research and Innovation reviewed the project as project number SDB 2024–018 (23–09–2024).

## CRediT authorship contribution statement

**Tariq A. Dam:** Writing – review & editing, Writing – original draft, Visualization, Validation, Software, Project administration, Methodology, Investigation, Formal analysis, Data curation, Conceptualization. **Daan de Bruin:** Writing – review & editing, Software, Resources, Formal analysis, Data curation, Conceptualization. **Giovanni Cinà:** Writing – review & editing, Software, Methodology, Formal analysis, Data curation, Conceptualization. **Patrick J. Thoral:** Conceptualization, Methodology, Supervision, Writing – original draft, Writing – review & editing. **Paul W.G. Elbers:** Writing – review & editing, Supervision, Formal analysis, Data curation, Conceptualization. **Corstiaan A. den Uil:** Writing – review & editing, Supervision, Formal analysis, Data curation, Conceptualization. **Reinier F. Crane:** Writing – review & editing, Supervision, Formal analysis, Data curation, Conceptualization.

## Conflict of Interest

Amsterdam University Medical Centers is entitled to royalties from Pacmed B.V. Pacmed B.V. develops and sells clinical implementations of artificial intelligence models. Santeon Working Group collaborates with Pacmed B.V. for the development of clinical artificial intelligence models. Tariq Dam was a part-time employee of Pacmed B.V.

## Appendix Supplementary materials

Image, application 1
