# Supplementary material for: ICU readmission and mortality risk prediction: Generalizability of a multi-hospital model
Source: J Intensive Med. 2025 Jun 14;5(4):377–84. doi: 10.1016/j.jointm.2025.03.007 (PMC12572862; doi:10.1016/j.jointm.2025.03.007)
Supplement: Supplementary file 1 [file mmc1.docx]

Supplemental material

Generalizability of ICU readmission risk prediction: A Multi-Hospital Model

# Figures


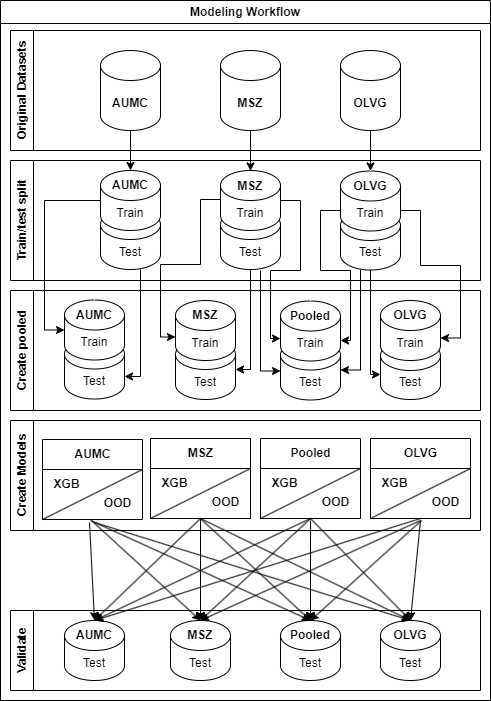


## Supplementary Figure S1. Modeling workflow detailing data flow, train/test splits and construction of the pooled dataset.

**
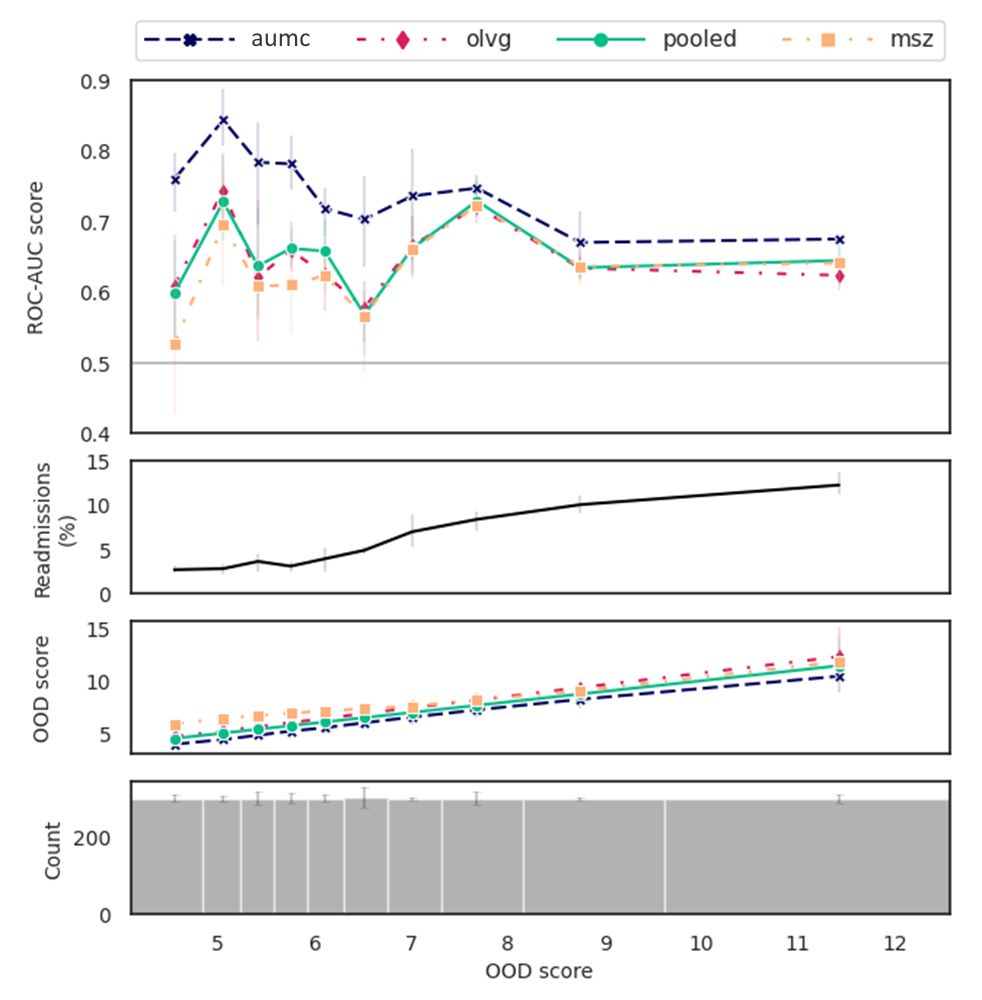
**

## Supplementary Figure S2. Model performance by AUROC score over OOD-score according to the Pooled model on the AUMC dataset, using 10 bins of equal sample size and 5-fold cross validation, along with the readmission rate and median OOD-score of each hospital. Detailed values of features and admission specialties are available in Supplementary Table 18, predicted probabilities are reported in Supplementary Figure S3, and aggregated patient characteristics over OOD scores are visualized in Supplementary Figure S4.


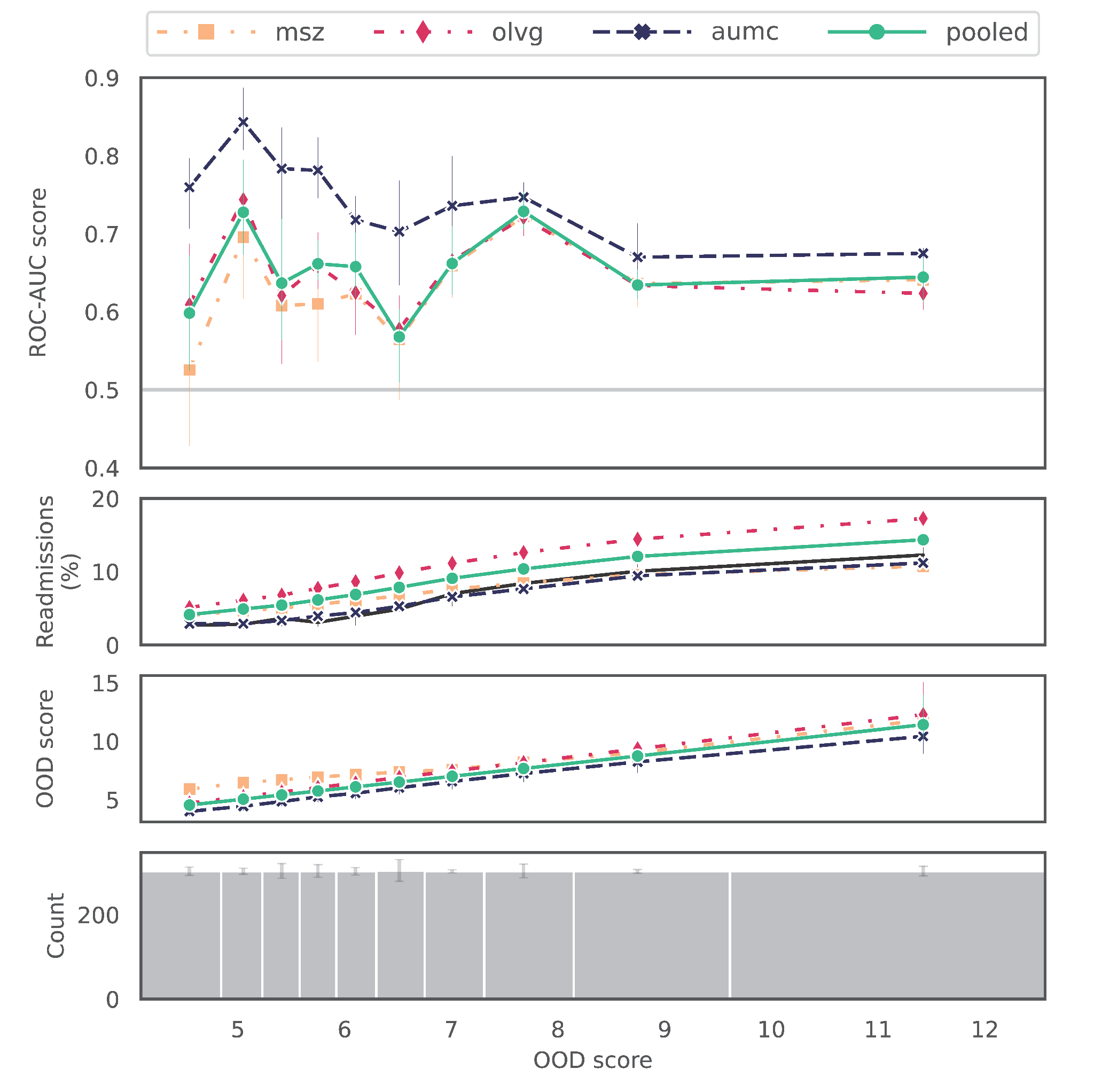


## Supplementary Figure S3. ROC-AUC score over OOD score with average predicted probabilities per prediction model reported alongside the actual readmission rate. This figure supplements supplementary figure S2.


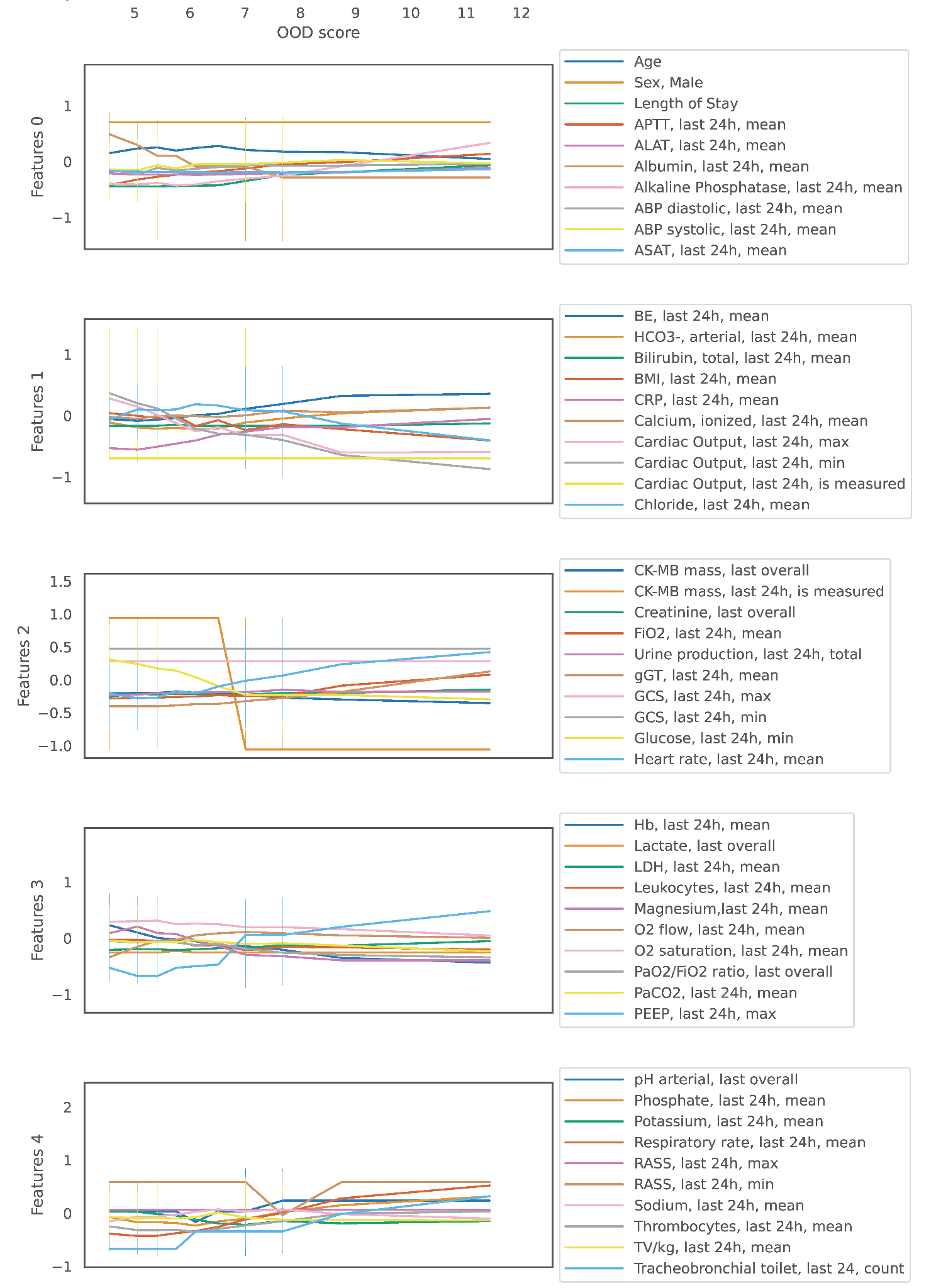

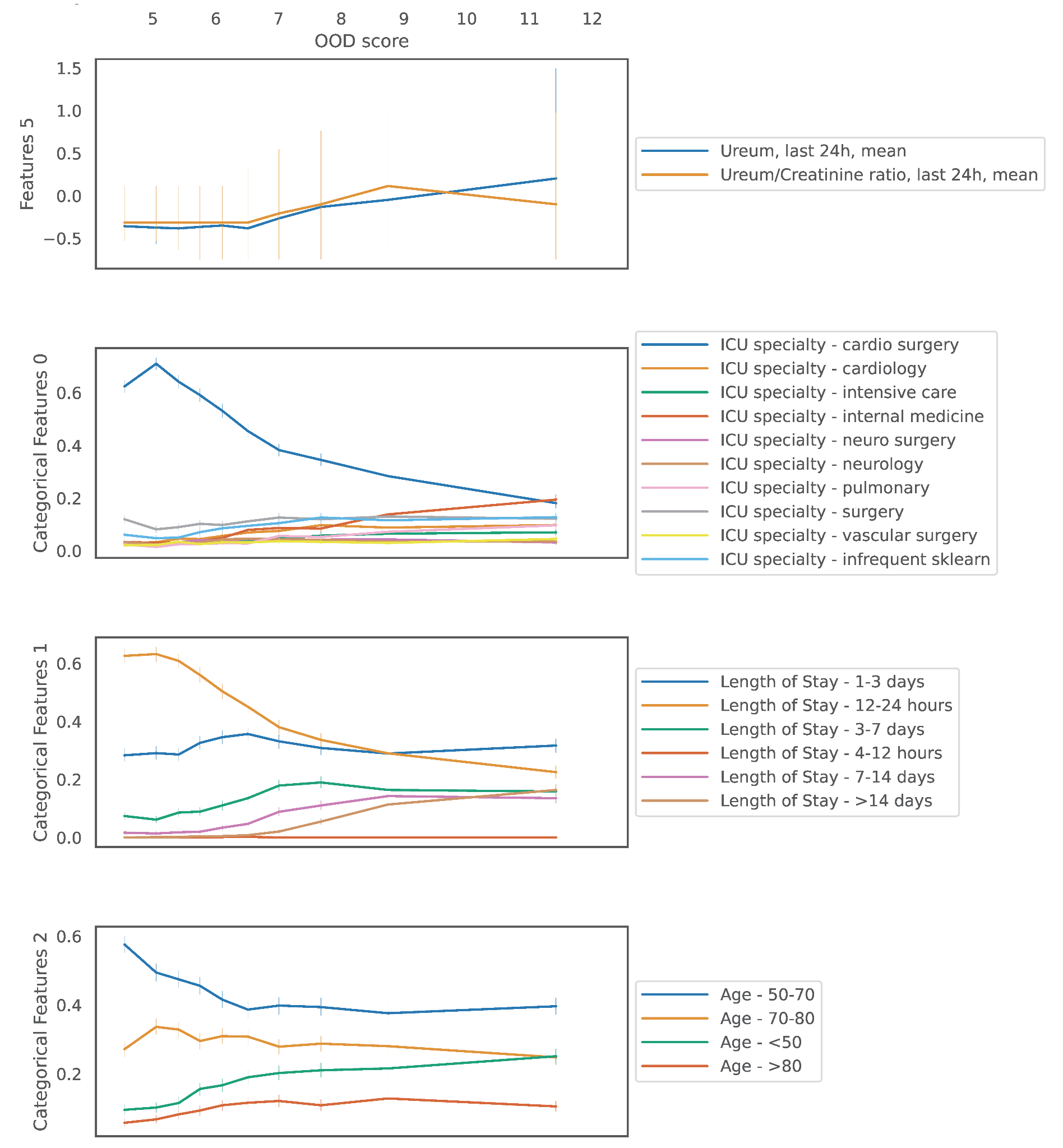


## Supplementary Figure S4. Patient characteristics averaged over OOD-score bins and folds on the AmsterdamUMC dataset. Values are standardized and scaled by subtracting the mean and scaling to one standard deviation. X-axis is shared with supplementary figures S2 and S3.


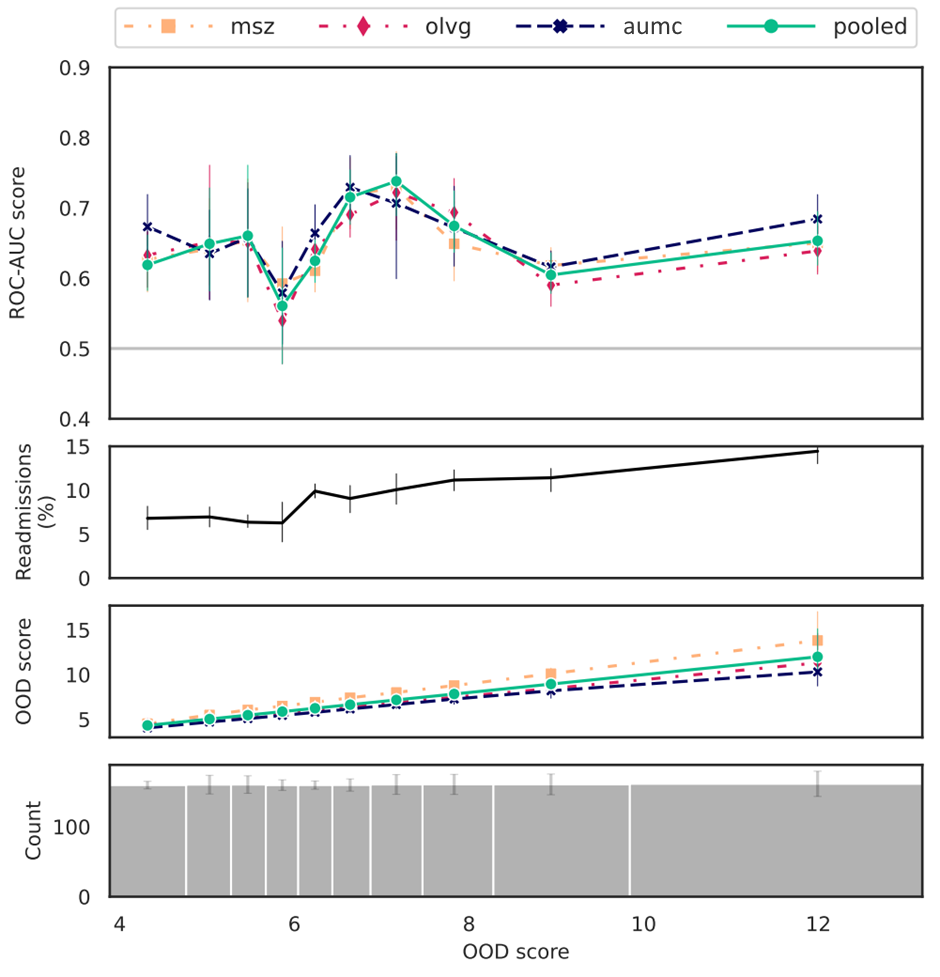


## Supplementary Figure S5. AUROC score over OOD score of the pooled model on AUMC data after exclusion of cardiac surgery patients for the sensitivity analysis. Predicted probabilities are reported in Supplementary Figure S6, and aggregated patient characteristics over OOD scores are visualized in Supplementary Figure S7.


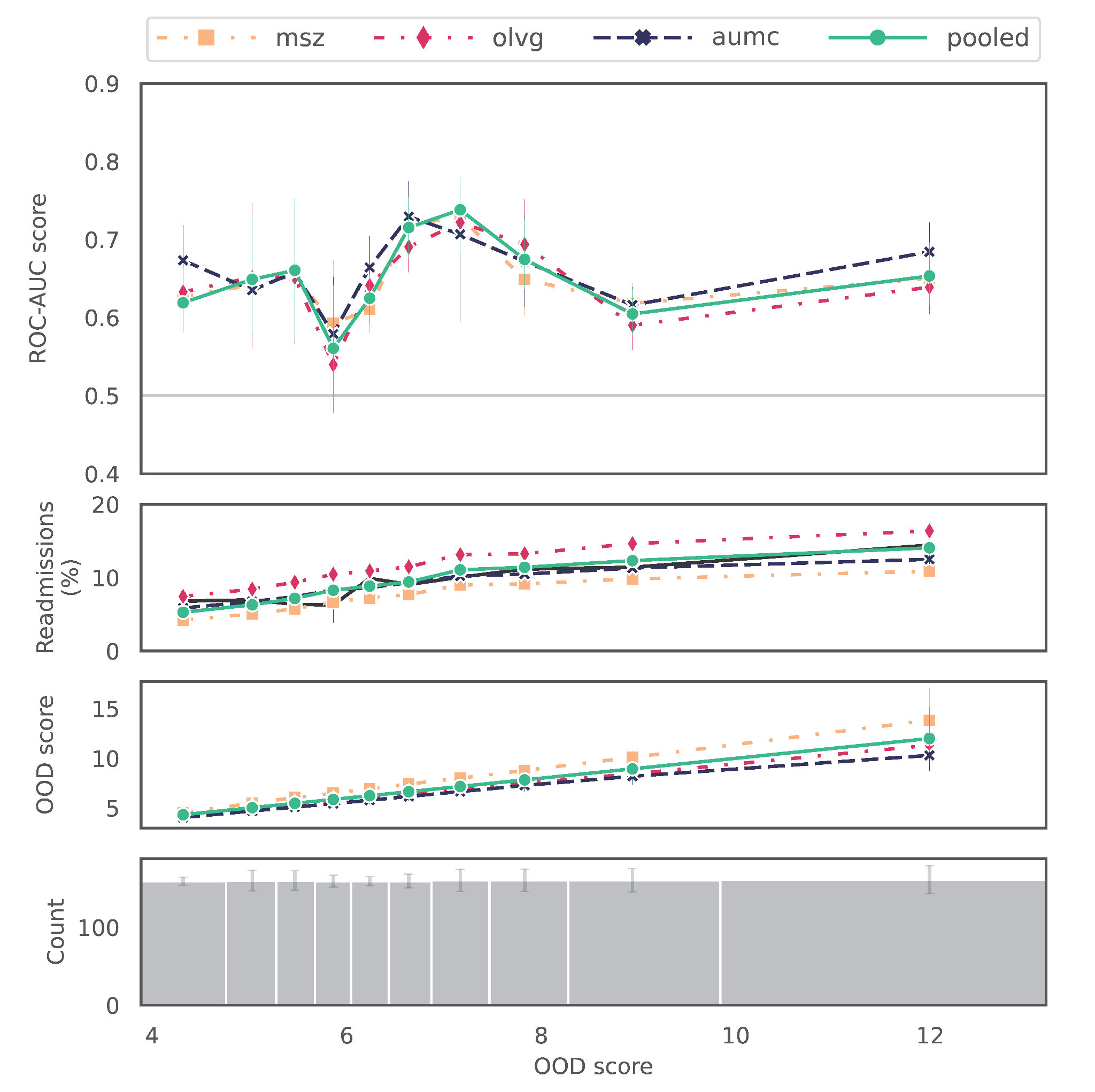


## Supplementary Figure S6. ROC-AUC score over OOD score with average predicted probabilities per prediction model reported alongside the actual readmission rate for AmsterdamUMC data after exclusion of cardiac surgery patients. This figure supplements supplementary figure S5 and S7.


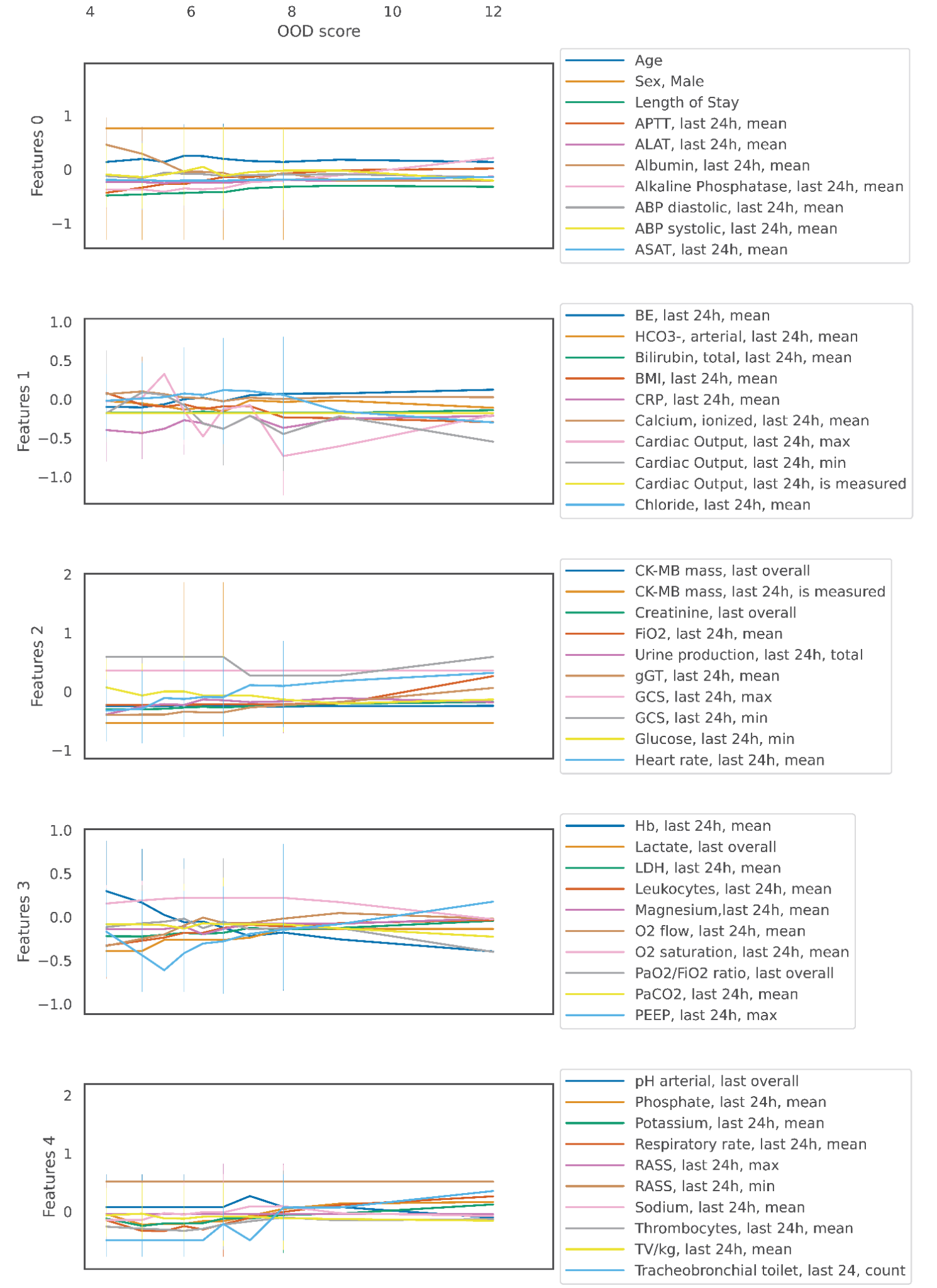


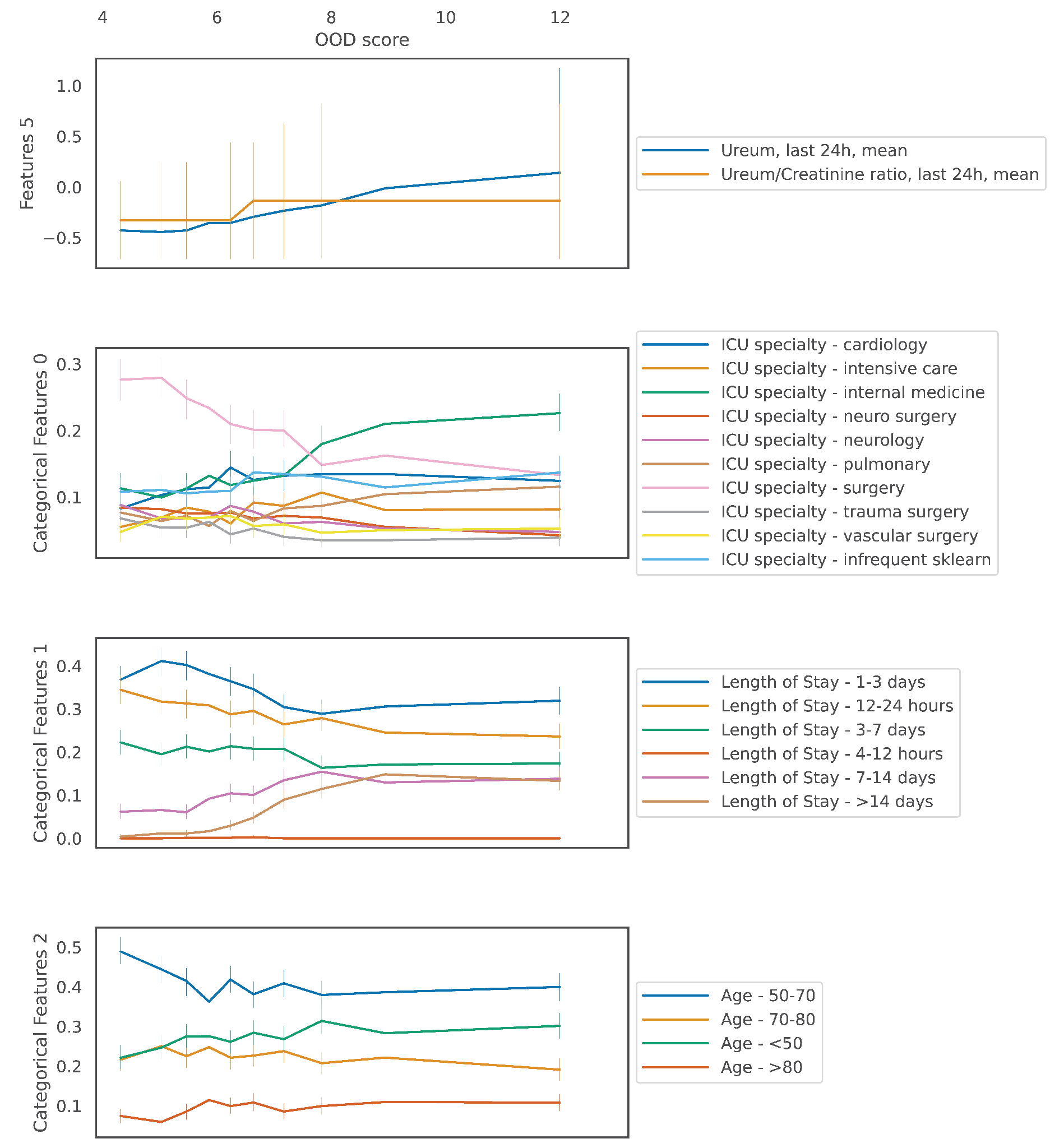


## Supplementary Figure S7. Patient characteristics averaged over OOD-score bins and folds on the AmsterdamUMC dataset subset after excluding cardiac surgery patients. Values are standardized and scaled by subtracting the mean and scaling to one standard deviation. X-axis is shared with supplementary figure S5 and S6.


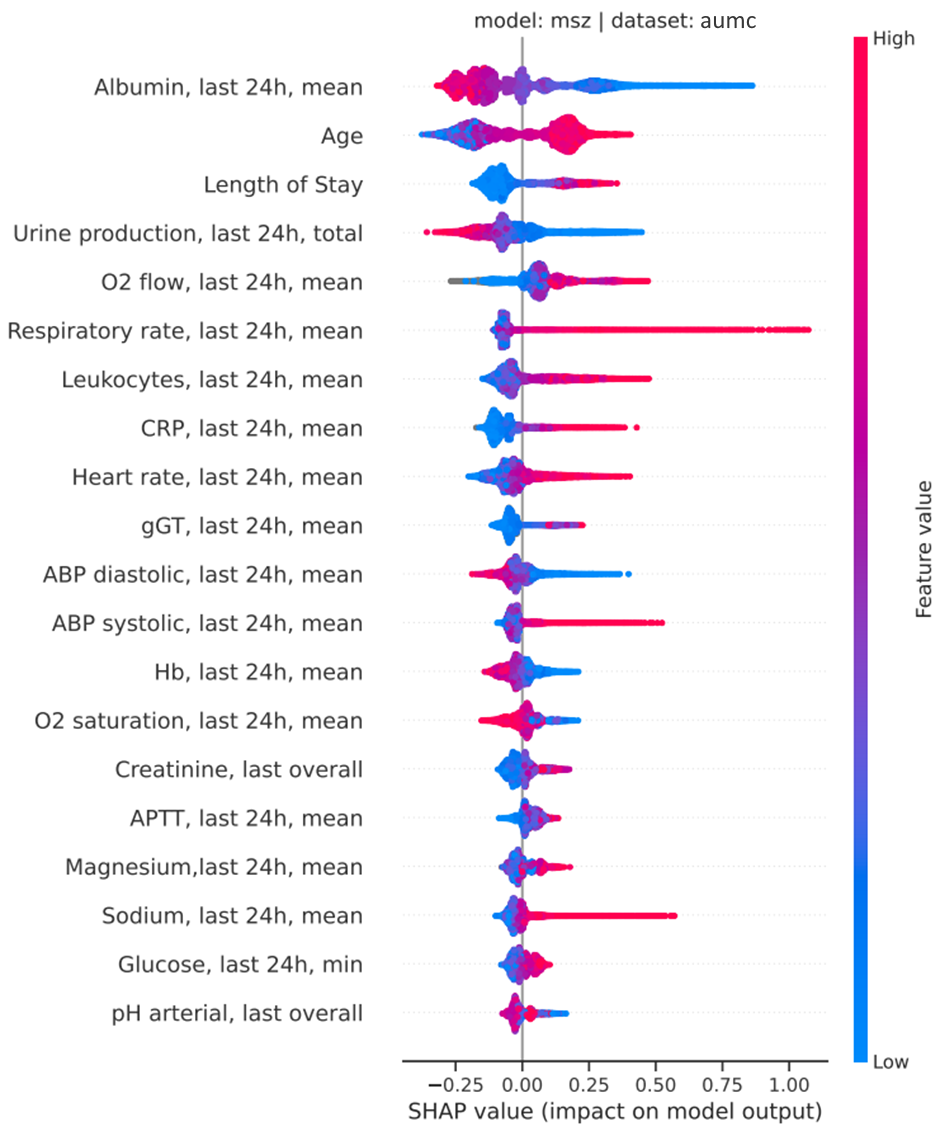


## Supplementary Figure S8. SHAP summary plot for MSZ model on AUMC data.


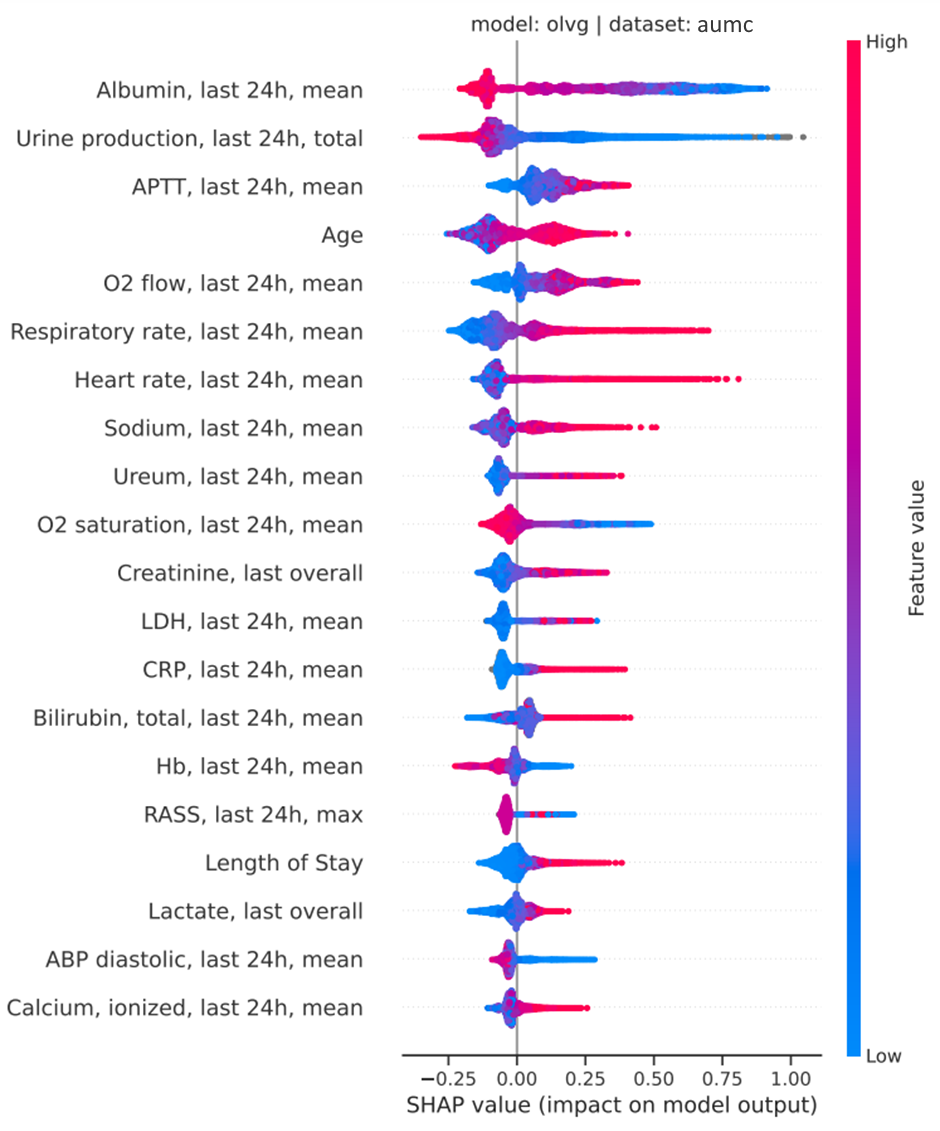


## Supplementary Figure S9. SHAP summary plot for OLVG model on AUMC data.


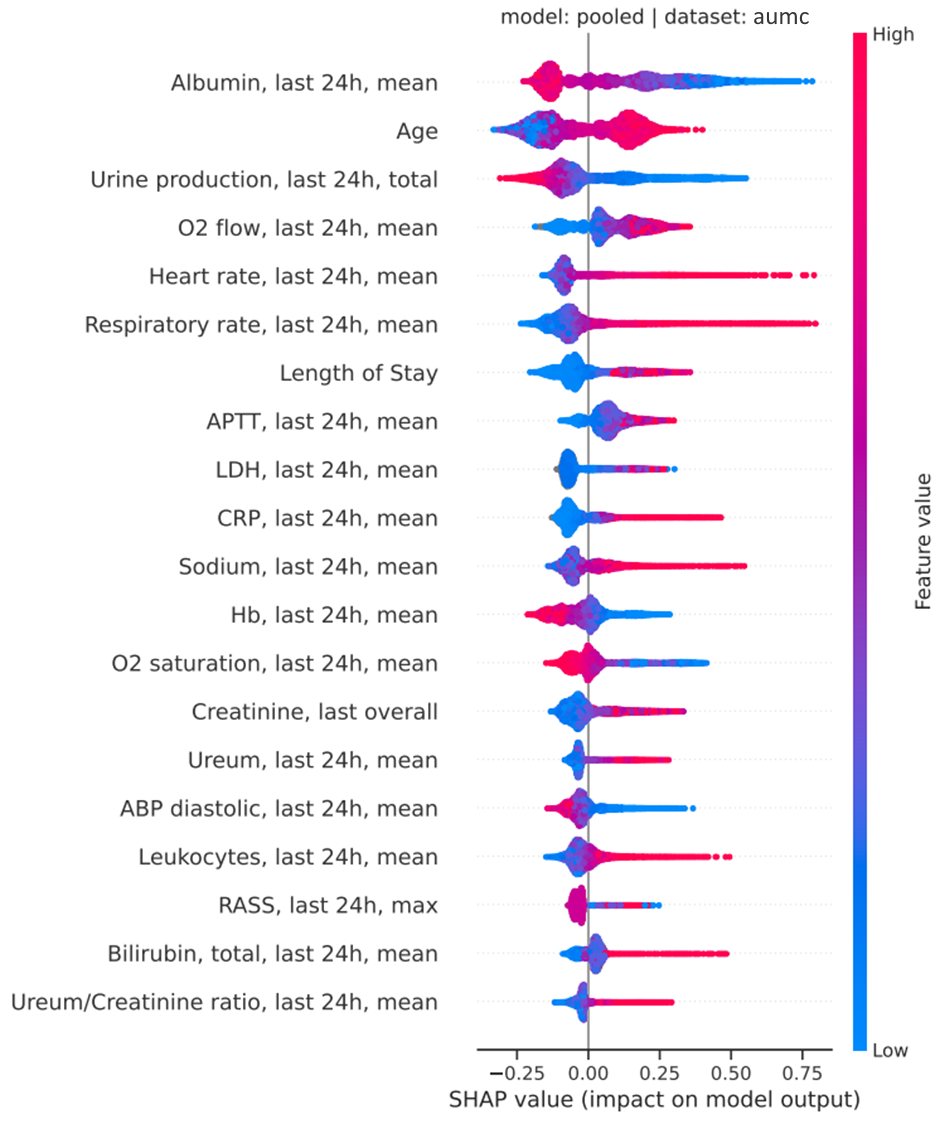


## Supplementary Figure S10. SHAP summary plot for Pooled model on AUMC data.


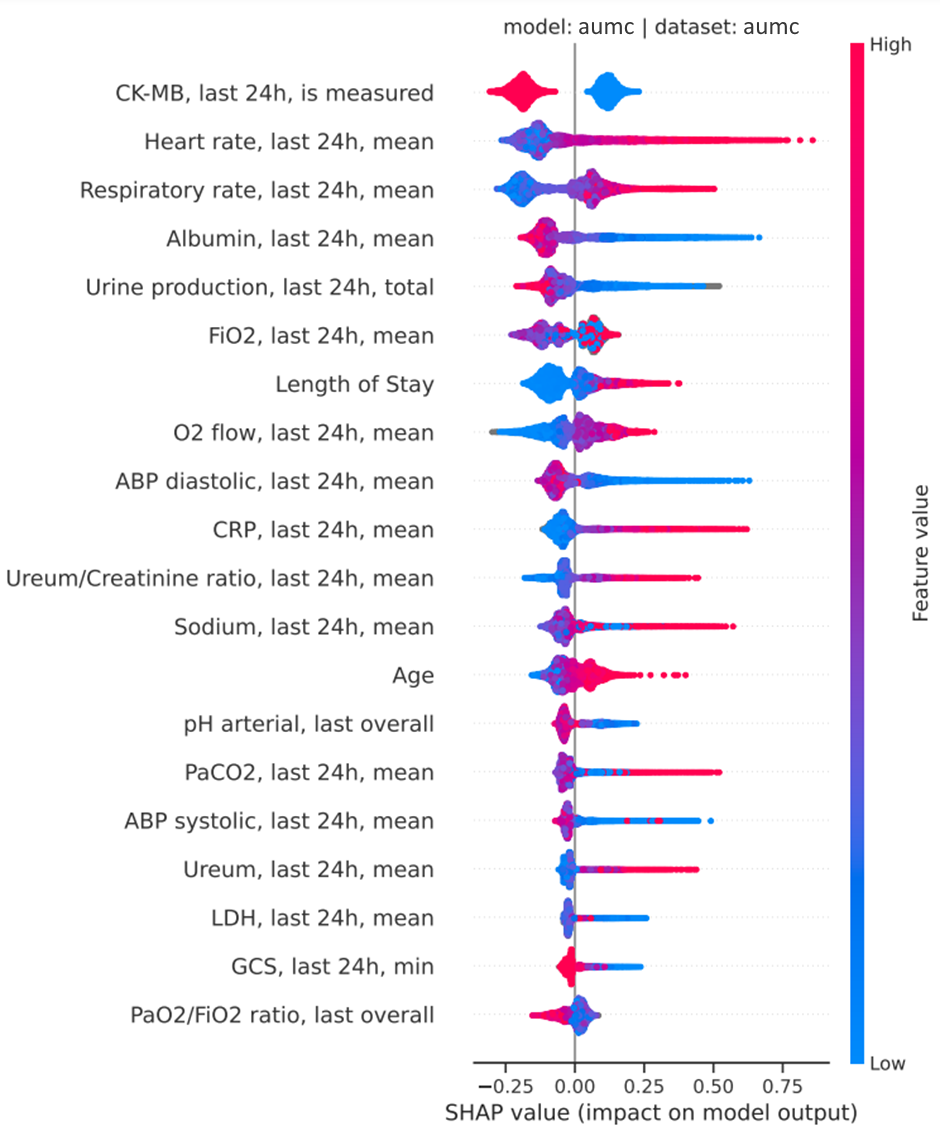


## Supplementary Figure S11. SHAP summary plot for AUMC model on AUMC data.


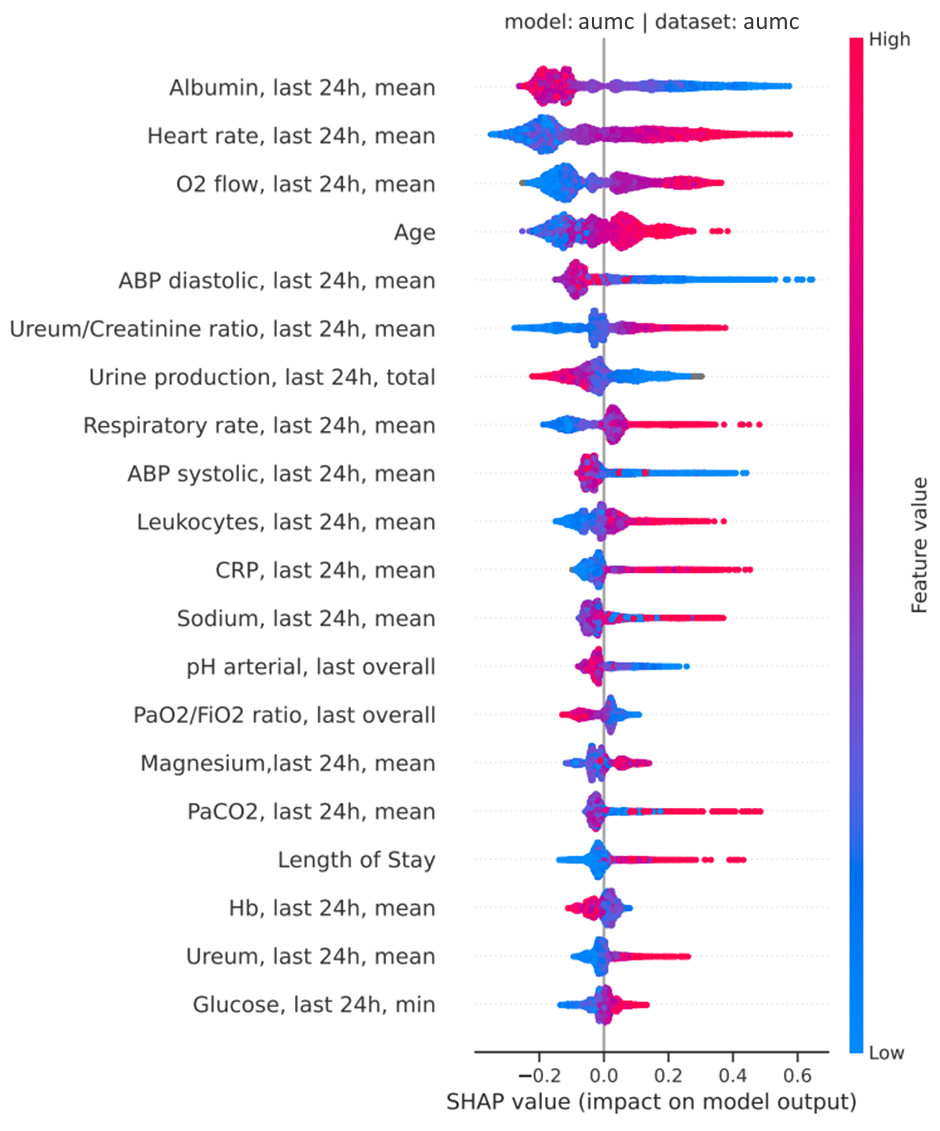


## Supplementary Figure S12. SHAP summary plot for the AUMC model on AUMC data after exclusion of cardiac surgery patients from both training and testing dataset.

# Tables

| **Name** | **Type** | **Aggregation** | **Window** | **Imputation strategy** |
| --- | --- | --- | --- | --- |
| **Age** | numeric | - | - | median |
| **Sex, Male** | bool | - | - | 0 |
| **Sex, Female** | bool | - | - | 0 |
| **APTT** | numeric | mean | last 24h | 35 |
| **ALAT** | numeric | mean | last 24h | median |
| **Albumin** | numeric | mean | last 24h | median |
| **Alkaline phosphatase** | numeric | mean | last 24h | median |
| **ABP diastolic** | numeric | mean | last 24h | median |
| **ABP systolic** | numeric | mean | last 24h | median |
| **ASAT** | numeric | mean | last 24h | median |
| **Base Excess** | numeric | mean | last 24h | median |
| **Bicarbonate, arterial** | numeric | mean | last 24h | median |
| **Bilirubin, total** | numeric | mean | last 24h | 10 |
| **BMI** | numeric | mean | last 24h | median |
| **CRP** | numeric | mean | last 24h | 4 |
| **Calcium, ionized** | numeric | mean | last 24h | median |
| **Cardiac output** | numeric | minimum | last 24h | median |
| **Cardiac output** | numeric | maximum | last 24h | median |
| **Cardiac output** | bool | is measured | last 24h | 0 |
| **Chloride** | numeric | mean | last 24h | median |
| **CK-MB** | numeric | last | overall | median |
| **CK-MB** | bool | is measured | last 24h | 0 |
| **Creatinine** | numeric | last | overall | median |
| **FiO2** | numeric | mean | last 24h | 21 |
| **Fluid out, urine** | numeric | total | last 24h | 0 |
| **Gamma-GT** | numeric | mean | last 24h | 55 |
| **GCS** | numeric | minimum | last 24h | 15 |
| **GCS** | numeric | maximum | last 24h | 15 |
| **Glucose** | numeric | minimum | last 24h | median |
| **Heart rate** | numeric | mean | last 24h | median |
| **Hemoglobin** | numeric | mean | last 24h | median |
| **Lactate** | numeric | last | overall | 1.1 |
| **LDH** | numeric | mean | last 24h | median |
| **Leukocytes** | numeric | mean | last 24h | 10 |
| **Magnesium** | numeric | mean | last 24h | 0.8 |
| **O2 flow** | numeric | mean | last 24h | 0 |
| **O2 saturation** | numeric | mean | last 24h | 0 |
| **PaO2/FiO2 ratio** | numeric | last | overall | median |
| **PCO2, arterial** | numeric | mean | last 24h | median |
| **PEEP** | numeric | maximum | last 24h | 0 |
| **pH, arterial** | numeric | last | overall | median |
| **Phosphate** | numeric | mean | last 24h | median |
| **Potassium** | numeric | mean | last 24h | median |
| **Respiratory rate** | numeric | mean | last 24h | median |
| **RASS** | numeric | minimum | last 24h | 0 |
| **RASS** | numeric | maximum | last 24h | 0 |
| **Sodium** | numeric | mean | last 24h | 141 |
| **Thrombocytes** | numeric | mean | last 24h | median |
| **Tidal volume / kg** | numeric | mean | last 24h | median |
| **Tracheobronchial toilet** | numeric | sum | last 24h | 0 |
| **Ureum** | numeric | mean | last 24h | median |
| **Ureum over Creatinine** | numeric | mean | last 24h | median |

## Supplementary Table S1. Features with their data type, aggregation type, window from the moment of discharge and imputation strategy or values. APTT = activated partial thromboplastin time, ALAT = alanine transaminase, ABP = arterial blood pressure, ASAT = aspartate transaminase, BMI = body mass index, CRP = c-reactive protein,

| **Model** | **Version** | **colsample bytree** | **gamma** | **learning rate** | **max depth** | **min child weight** | **n estimators** | **objective** | **reg lambda** | **subsample** | **n jobs** | **random state** |
| --- | --- | --- | --- | --- | --- | --- | --- | --- | --- | --- | --- | --- |
| XGBoost | 1.7.6 | 0.8 | 5 | 0.005 | 5 | 1.5 | 1300 | binary:logistic | 10 | 0.4 | -1 | 42 |

## Supplementary Table S2. Hyperparameters used for fitting the prediction models. Hyperparameters were kept consistent across datasets and trained models. Optimal hyperparameters were derived from prior product implementation and kept consistent for comparison.

| **Package** | **Version** | **Model** | **hidden neurons** | **epochs** | **random_state** |
| --- | --- | --- | --- | --- | --- |
| Pyod | 1.1.2 | AutoEncoder | [25, 25, 25, 10, 10, 10, 25, 25, 25] | 10 | 42 |

## Supplementary Table S3. Settings for training the Out of Domain model based on variational autoencoder.

| **Parameter** | **Value** | **Missing, N (%)** |
| --- | --- | --- |
| **Age, years, Median [IQR]** | 66.67 [56.85, 74.17] | 0 (0.0%) |
| **Age, categories, N (%)** |  |  |
| **- <50 years** | 6744 (15.04%) | 0 (0.0%) |
| **- 50-70 years** | 20591 (45.92%) | 0 (0.0%) |
| **- 70-80 years** | 13270 (29.6%) | 0 (0.0%) |
| **- >80 years** | 4232 (9.44%) | 0 (0.0%) |
| **Sex, N (%)** |  |  |
| **- Female** | 15114 (33.71%) | 289 (0.64%) |
| **- Male** | 29434 (65.65%) | 289 (0.64%) |
| **BMI, mean, last 24h, Median [IQR]** | 26.25 [23.67, 29.41] | 18464.0 (41.18%) |
| **Hospital, name, N (%)** |  |  |
| **- MSZ** | 10092 (22.51%) |  |
| **- OLVG** | 19417 (43.31%) |  |
| **- AUMC** | 15328 (34.19%) |  |
| **ICU specialty, N (%)** |  |  |
| **- Cardiac surgery** | 18765 (41.85%) |  |
| **- Unknown** | 10095 (22.51%) |  |
| **- Surgery** | 4103 (9.15%) |  |
| **- Internal medicine** | 2954 (6.59%) |  |
| **- Cardiology** | 2208 (4.92%) |  |
| **- Pulmonary** | 1970 (4.39%) |  |
| **- Neurology** | 793 (1.77%) |  |
| **- ICU** | 646 (1.44%) |  |
| **- Neurosurgery** | 571 (1.27%) |  |
| **- Gastroenterology** | 543 (1.21%) |  |
| **- Vascular surgery** | 477 (1.06%) |  |
| **- Other** | 1712 (3.82%) |  |
| **Length of Stay, hours, Median [IQR]** | 26.33 [20.62, 69.0] |  |
| **Length of Stay, N (%)** |  |  |
| **- 4-12 hours** | 12 (0.03%) |  |
| **- 12-24 hours** | 19767 (44.09%) |  |
| **- 1-3 days** | 14555 (32.46%) |  |
| **- 3-7 days** | 5993 (13.37%) |  |
| **- 7-14 days** | 2609 (5.82%) |  |
| **>14 days** | 1901 (4.24%) |  |
| ***Vitals*** |  |  |
| **Respiratory rate, mean, last 24h, Median [IQR]** | 17.12 [14.75, 20.15] | 1655.0 (3.69%) |
| **Tidal volume/kg, mean, last 24h, Median [IQR]** | 7.39 [6.6, 8.3] | 15007.0 (33.47%) |
| **O2 flow, mean, last 24h, Median [IQR]** | 3.0 [1.96, 4.53] | 6255.0 (13.95%) |
| **FiO2, mean, last 24h, Median [IQR]** | 40.0 [31.25, 42.31] | 23937.0 (53.39%) |
| **O2 saturation, mean, last 24h, Median [IQR]** | 96.58 [95.17, 97.86] | 661.0 (1.47%) |
| **Tracheobronchial toilet, count, last 24h, Median [IQR]** | 1.0 [0.0, 2.0] | 29771.0 (66.4%) |
| **PEEP, maximum, last 24h, Median [IQR]** | 8.16 [6.12, 10.0] | 12953.0 (28.89%) |
| **Heart rate, mean, last 24h, Median [IQR]** | 80.92 [72.41, 90.67] | 126.0 (0.28%) |
| **ABP systolic, mean, last 24h, Median [IQR]** | 123.09 [111.44, 136.85] | 2495.0 (5.56%) |
| **ABP diastolic, mean, last 24h, Median [IQR]** | 60.82 [54.92, 68.0] | 2495.0 (5.56%) |
| **Cardiac output, maximum, last 24h, Median [IQR]** | 6.1 [5.1, 7.2] | 39170.0 (87.36%) |
| **Cardiac output, minimum, last 24h, Median [IQR]** | 4.5 [3.7, 5.4] | 39170.0 (87.36%) |
| **Cardiac output, is measured, last 24h, N (%)** | 5667 (12.64%) | 0.0 (0.0%) |
| **Fluid out, urine, total, last 24h, Median [IQR]** | 1720.67 [1191.44, 2465.0] | 496.0 (1.11%) |
| **GCS, maximum, last 24h, Median [IQR]** | 15.0 [15.0, 15.0] | 21601.0 (48.18%) |
| **GCS, minimum, last 24h, Median [IQR]** | 15.0 [13.0, 15.0] | 21601.0 (48.18%) |
| **RASS, maximum, last 24h, Median [IQR]** | 0.0 [0.0, 0.0] | 11987.0 (26.73%) |
| **RASS, minimum, last 24h, Median [IQR]** | -1.0 [-3.0, 0.0] | 11987.0 (26.73%) |
| ***Lab*** |  |  |
| **CRP, mean, last 24h, Median [IQR]** | 51.0 [22.0, 107.0] | 17138.0 (38.22%) |
| **Hb, mean, last 24h, Median [IQR]** | 6.6 [5.9, 7.45] | 1150.0 (2.56%) |
| **Leukocytes, mean, last 24h, Median [IQR]** | 12.65 [9.6, 16.3] | 4295.0 (9.58%) |
| **Thrombocytes, mean, last 24h, Median [IQR]** | 192.67 [145.0, 261.0] | 3281.0 (7.32%) |
| **APTT, mean, last 24h, Median [IQR]** | 29.7 [25.5, 37.0] | 5200.0 (11.6%) |
| **Sodium, mean, last 24h, Median [IQR]** | 138.33 [136.25, 140.5] | 2071.0 (4.62%) |
| **Chloride, mean, last 24h, Median [IQR]** | 106.8 [104.0, 109.33] | 5888.0 (13.13%) |
| **Potassium, mean, last 24h, Median [IQR]** | 4.12 [3.9, 4.4] | 2022.0 (4.51%) |
| **Magnesium, mean, last 24h, Median [IQR]** | 0.91 [0.8, 1.07] | 4673.0 (10.42%) |
| **Phosphate, mean, last 24h, Median [IQR]** | 1.05 [0.87, 1.24] | 4171.0 (9.3%) |
| **Calcium ionized, mean, last 24h, Median [IQR]** | 1.16 [1.12, 1.2] | 8561.0 (19.09%) |
| **pH, arterial, last, overall, Median [IQR]** | 7.43 [7.39, 7.46] | 3293.0 (7.34%) |
| **PaO2/FiO2 ratio, last, overall, Median [IQR]** | 257.5 [197.5, 336.67] | 12275.0 (27.38%) |
| **PCO2 arterial, mean, last 24h, Median [IQR]** | 39.0 [35.78, 42.5] | 5262.0 (11.74%) |
| **Base Excess, mean, last 24h, Median [IQR]** | 0.76 [-1.33, 2.66] | 4002.0 (8.93%) |
| **Bicarbonate arterial, mean, last 24h, Median [IQR]** | 24.13 [22.4, 26.27] | 5339.0 (11.91%) |
| **Lactate, last, overall, Median [IQR]** | 1.3 [0.9, 1.8] | 10530.0 (23.49%) |
| **Glucose, minimum, last 24h, Median [IQR]** | 6.4 [5.5, 7.4] | 1839.0 (4.1%) |
| **LDH, mean, last 24h, Median [IQR]** | 251.0 [191.0, 345.0] | 26820.0 (59.82%) |
| **CK-MB, last, overall, Median [IQR]** | 12.6 [5.5, 23.5] | 31260.0 (69.72%) |
| **CK-MB, is measured, last 24h, N (%)** | 8758 (20.07%) | 1207.0 (2.69%) |
| **Creatinine, last, overall, Median [IQR]** | 75.0 [59.0, 99.0] | 1833.0 (4.09%) |
| **Ureum, mean, last 24h, Median [IQR]** | 7.5 [5.3, 11.6] | 19812.0 (44.19%) |
| **Ureum/creatinine ratio, mean, last 24h, Median [IQR]** | 0.1 [0.07, 0.13] | 19886.0 (44.35%) |
| **ASAT, mean, last 24h, Median [IQR]** | 37.0 [24.0, 66.0] | 36382.0 (81.14%) |
| **ALAT, mean, last 24h, Median [IQR]** | 30.0 [18.0, 58.0] | 20324.0 (45.33%) |
| **Alkaline phosphatase, mean, last 24h, Median [IQR]** | 75.0 [55.0, 109.62] | 37081.0 (82.7%) |
| **gammaGT, mean, last 24h, Median [IQR]** | 42.5 [21.0, 104.0] | 30150.0 (67.24%) |
| **Bilirubin total, mean, last 24h, Median [IQR]** | 8.0 [6.0, 12.0] | 19697.0 (43.93%) |
| **Albumin, mean, last 24h, Median [IQR]** | 26.2 [22.0, 31.0] | 9254.0 (20.64%) |
| ***Outcomes*** |  |  |
| **Readmission <7d, N (%)** | 2494 (5.56%) |  |
| **Readmission or mortality <7d, N (%)** | 2967 (6.62%) |  |
| **Mortality, N (%)** |  |  |
| **- 7 day** | 629 (1.4%) |  |
| **- 30 day** | 1500 (3.35%) |  |
| **- 90 day** | 2135 (4.76%) |  |

## Supplementary Table S4. Patient characteristics for all hospitals combined, before imputation.

|  |  | **Value** | | |
| --- | --- | --- | --- | --- |
| **Feature** | **Description** | **MSZ** | **OLVG** | **AUMC** |
| **Patients** | **N (%)** | 9959 (100.0%) | 19221 (100.0%) | 15134 (100.0%) |
| **Age** | **Median [IQR]** | 65.0 [54.0, 74.0] | 67.0 [58.0, 74.0] | 66.6 [56.0, 74.93] |
| **Age, <50** | **N (%)** | 1932 (19.4%) | 2143 (11.15%) | 2577 (17.03%) |
| **Age, 50-70** | **N (%)** | 4610 (46.29%) | 9114 (47.42%) | 6610 (43.68%) |
| **Age, 70-80** | **N (%)** | 2547 (25.57%) | 6115 (31.81%) | 4454 (29.43%) |
| **Age, >80** | **N (%)** | 870 (8.74%) | 1849 (9.62%) | 1493 (9.87%) |
| **Sex, Male** | **N (%)** | 5987 (60.12%) | 12986 (67.56%) | 10111 (66.81%) |
| **BMI, last 24h, mean** | **Median [IQR]** | 26.42 [23.53, 30.86] | 26.53 [23.84, 29.73] | 25.74 [23.51, 28.37] |
| **Length of Stay** | **Median [IQR]** | 41.27 [21.3, 85.7] | 24.5 [19.67, 59.32] | 24.68 [21.12, 65.87] |
| **Length of Stay, 4-12 hours** | **N (%)** |  | 7 (0.04%) | 5 (0.03%) |
| **Length of Stay, 12-24 hours** | **N (%)** | 3436 (34.5%) | 9347 (48.63%) | 6984 (46.15%) |
| **Length of Stay, 1-3 days** | **N (%)** | 3727 (37.42%) | 6082 (31.64%) | 4746 (31.36%) |
| **Length of Stay, 3-7 days** | **N (%)** | 1700 (17.07%) | 2405 (12.51%) | 1888 (12.48%) |
| **Length of Stay, 7-14 days** | **N (%)** | 723 (7.26%) | 939 (4.89%) | 947 (6.26%) |
| **Length of Stay, >14 days** | **N (%)** | 373 (3.75%) | 441 (2.29%) | 564 (3.73%) |
| **Readmission/Mortality after discharge, 7d** | **N (%)** | 706 (7.09%) | 1321 (6.87%) | 891 (5.89%) |
| **Readmission after discharge, 7d** | **N (%)** | 577 (5.79%) | 1153 (6.0%) | 722 (4.77%) |
| **Mortality, 7d** | **N (%)** | 175 (1.76%) | 228 (1.19%) | 218 (1.44%) |
| **Mortality, 30d** | **N (%)** | 476 (4.78%) | 523 (2.72%) | 477 (3.15%) |
| **Mortality, 90d** | **N (%)** | 775 (7.78%) | 658 (3.42%) | 649 (4.29%) |
| **GCS, last 24h, max** | **Median [IQR]** | 15.0 [15.0, 15.0] | 8.0 [3.0, 15.0] | 15.0 [15.0, 15.0] |
| **GCS, last 24h, min** | **Median [IQR]** | 15.0 [14.0, 15.0] | 3.0 [3.0, 13.0] | 15.0 [14.0, 15.0] |
| **RASS, last 24h, max** | **Median [IQR]** | 0.0 [0.0, 0.0] | 0.0 [0.0, 0.0] | 0.0 [0.0, 0.0] |
| **RASS, last 24h, min** | **Median [IQR]** | -1.0 [-1.0, 0.0] | -2.0 [-4.0, -1.0] | 0.0 [-1.0, 0.0] |
| **Heart rate, last 24h, mean** | **Median [IQR]** | 81.23 [71.28, 91.75] | 80.85 [72.96, 90.29] | 80.54 [72.05, 90.05] |
| **ABP systolic, last 24h, mean** | **Median [IQR]** | 127.33 [114.55, 142.15] | 118.92 [108.52, 131.91] | 126.09 [114.1, 138.59] |
| **ABP diastolic, last 24h, mean** | **Median [IQR]** | 63.11 [55.7, 72.14] | 59.14 [53.83, 65.47] | 61.67 [56.11, 68.38] |
| **Respiratory rate, last 24h, mean** | **Median [IQR]** | 18.17 [15.9, 20.91] | 16.8 [14.29, 20.0] | 16.55 [14.62, 19.45] |
| **O2 saturation, last 24h, mean** | **Median [IQR]** | 97.04 [95.78, 98.16] | 96.27 [95.06, 97.48] | 96.67 [94.86, 98.0] |
| **Tracheobronchial toilet, last 24, count** | **Median [IQR]** | 2.0 [1.0, 3.0] | 1.0 [0.0, 2.0] | 2.0 [1.0, 4.0] |
| **O2 flow, last 24h, mean** | **Median [IQR]** | 2.0 [1.55, 3.82] | 2.33 [1.64, 3.43] | 4.42 [3.0, 5.08] |
| **FiO2, last 24h, mean** | **Median [IQR]** | 40.0 [32.99, 46.67] | 34.44 [27.25, 40.0] | 40.43 [39.98, 45.07] |
| **PEEP, last 24h, max** | **Median [IQR]** | 10.0 [7.8, 12.1] | 8.16 [8.16, 9.18] | 6.4 [5.2, 9.4] |
| **TV/kg, last 24h, mean** | **Median [IQR]** | 7.68 [6.91, 8.61] | 7.56 [6.7, 8.47] | 7.18 [6.45, 8.04] |
| **PaO2/FiO2 ratio, last overall** | **Median [IQR]** | 235.0 [183.62, 308.57] | 253.33 [191.43, 331.49] | 267.5 [207.5, 350.0] |
| **PaCO2, last 24h, mean** | **Median [IQR]** | 38.17 [34.5, 42.29] | 39.67 [36.0, 43.0] | 39.0 [36.0, 42.0] |
| **pH arterial, last overall** | **Median [IQR]** | 7.43 [7.4, 7.46] | 7.43 [7.4, 7.46] | 7.42 [7.39, 7.45] |
| **BE, last 24h, mean** | **Median [IQR]** | 0.5 [-1.67, 2.83] | 0.2 [-1.5, 2.33] | 1.29 [-0.75, 2.81] |
| **HCO3-, arterial, last 24h, mean** | **Median [IQR]** | 24.71 [22.5, 27.0] | 24.42 [22.8, 26.35] | 23.48 [21.98, 25.43] |
| **Lactate, last overall** | **Median [IQR]** | 1.3 [0.9, 1.8] | 1.3 [0.9, 1.8] | 1.2 [0.9, 1.7] |
| **Glucose, last 24h, min** | **Median [IQR]** | 6.1 [5.3, 7.1] | 6.6 [5.7, 7.6] | 6.4 [5.4, 7.4] |
| **ALAT, last 24h, mean** | **Median [IQR]** | 27.0 [16.0, 54.0] | 32.0 [20.0, 61.0] | 29.0 [18.0, 60.0] |
| **ASAT, last 24h, mean** | **Median [IQR]** | 31.0 [23.0, 56.0] | 41.0 [24.0, 83.0] | 40.0 [25.0, 68.0] |
| **Alkaline Phosphatase, last 24h, mean** | **Median [IQR]** | 73.0 [56.0, 99.0] | 86.0 [61.0, 127.0] | 72.0 [53.0, 108.0] |
| **gGT, last 24h, mean** | **Median [IQR]** | 41.0 [21.0, 99.0] | 54.0 [24.0, 127.0] | 41.0 [20.0, 99.0] |
| **Bilirubin, total, last 24h, mean** | **Median [IQR]** | 8.5 [6.0, 12.0] | 8.0 [5.0, 12.0] | 8.0 [6.0, 12.0] |
| **LDH, last 24h, mean** | **Median [IQR]** | 222.0 [182.0, 306.0] | 254.0 [192.0, 345.0] | 263.0 [196.0, 368.0] |
| **Albumin, last 24h, mean** | **Median [IQR]** | 25.0 [21.0, 30.0] | 30.0 [27.0, 33.5] | 24.5 [21.0, 28.0] |
| **APTT, last 24h, mean** | **Median [IQR]** | 29.0 [26.0, 33.0] | 25.9 [23.7, 28.95] | 38.0 [34.0, 43.0] |
| **Cardiac Output, last 24h, is measured** | **N (%)** | 30 (0.3%) | 691 (3.6%) | 4946 (32.68%) |
| **Cardiac Output, last 24h, max** | **Median [IQR]** | 6.78 [5.54, 8.81] | 7.3 [6.0, 9.1] | 5.9 [5.0, 7.0] |
| **Cardiac Output, last 24h, min** | **Median [IQR]** | 5.3 [3.85, 6.16] | 4.8 [3.9, 5.9] | 4.5 [3.7, 5.3] |
| **CK-MB, last 24h, is measured** | **N (%)** | 710 (7.13%) | 10939 (56.91%) | 8010 (52.93%) |
| **Creatinine, last overall** | **Median [IQR]** | 69.0 [52.0, 98.0] | 77.0 [62.0, 101.0] | 77.0 [60.0, 98.0] |
| **Ureum, last 24h, mean** | **Median [IQR]** | 6.55 [4.7, 10.0] | 9.0 [6.29, 13.5] | 6.6 [4.85, 9.5] |
| **Ureum/Creatinine ratio, last 24h, mean** | **Median [IQR]** | 0.09 [0.06, 0.12] | 0.11 [0.08, 0.15] | 0.08 [0.06, 0.11] |
| **Urine production, last 24h, total** | **Median [IQR]** | 1458.34 [967.6, 2198.33] | 1887.87 [1321.99, 2667.5] | 1686.38 [1200.0, 2366.98] |
| **Hb, last 24h, mean** | **Median [IQR]** | 6.8 [6.0, 7.75] | 6.4 [5.73, 7.29] | 6.72 [6.11, 7.48] |
| **Leukocytes, last 24h, mean** | **Median [IQR]** | 11.1 [8.6, 14.4] | 14.15 [10.5, 17.9] | 12.5 [9.7, 15.66] |
| **Thrombocytes, last 24h, mean** | **Median [IQR]** | 225.0 [168.0, 307.0] | 187.0 [144.0, 247.0] | 178.5 [136.0, 243.5] |
| **CRP, last 24h, mean** | **Median [IQR]** | 63.0 [24.08, 131.0] | 49.0 [23.0, 91.0] | 46.0 [20.0, 104.0] |
| **Sodium, last 24h, mean** | **Median [IQR]** | 138.0 [135.67, 140.5] | 138.25 [136.33, 140.33] | 138.62 [136.55, 140.8] |
| **Potassium, last 24h, mean** | **Median [IQR]** | 4.0 [3.8, 4.23] | 4.2 [3.97, 4.46] | 4.12 [3.9, 4.38] |
| **Chloride, last 24h, mean** | **Median [IQR]** | 105.75 [103.0, 108.5] | 107.0 [104.5, 109.33] | 107.0 [104.0, 109.89] |
| **Calcium, ionized, last 24h, mean** | **Median [IQR]** | 1.13 [1.1, 1.17] | 1.17 [1.12, 1.21] | 1.17 [1.14, 1.21] |
| **Phosphate, last 24h, mean** | **Median [IQR]** | 1.07 [0.89, 1.27] | 1.06 [0.89, 1.25] | 1.01 [0.83, 1.2] |
| **Magnesium,last 24h, mean** | **Median [IQR]** | 0.86 [0.78, 0.97] | 0.95 [0.82, 1.13] | 0.92 [0.79, 1.07] |
| **ICU Specialty, Anesthesiology** | **N (%)** |  |  | 2 (0.01%) |
| **ICU Specialty, Cardiac surgery** | **N (%)** |  | 11512 (59.89%) | 7179 (47.44%) |
| **ICU Specialty, Cardiology** | **N (%)** |  | 1222 (6.36%) | 961 (6.35%) |
| **ICU Specialty, ENT** | **N (%)** |  | 48 (0.25%) | 308 (2.04%) |
| **ICU Specialty, Gastroenterology** | **N (%)** |  | 357 (1.86%) | 167 (1.1%) |
| **ICU Specialty, Gynecology** | **N (%)** |  | 87 (0.45%) | 134 (0.89%) |
| **ICU Specialty, Hematology** | **N (%)** |  | 36 (0.19%) |  |
| **ICU Specialty, Intensive care** | **N (%)** |  |  | 632 (4.18%) |
| **ICU Specialty, Internal medicine** | **N (%)** |  | 1746 (9.08%) | 1152 (7.61%) |
| **ICU Specialty, MKA** | **N (%)** |  | 28 (0.15%) | 28 (0.19%) |
| **ICU Specialty, Neuro surgery** | **N (%)** |  |  | 557 (3.68%) |
| **ICU Specialty, Neurology** | **N (%)** |  | 230 (1.2%) | 542 (3.58%) |
| **ICU Specialty, Oncology** | **N (%)** |  | 12 (0.06%) | 32 (0.21%) |
| **ICU Specialty, Ophthalmology** | **N (%)** |  |  | 5 (0.03%) |
| **ICU Specialty, Orthopedic surgery** | **N (%)** |  | 136 (0.71%) | 106 (0.7%) |
| **ICU Specialty, Other** | **N (%)** |  | 5 (0.03%) | 27 (0.18%) |
| **ICU Specialty, Plastic surgery** | **N (%)** |  | 7 (0.04%) | 22 (0.15%) |
| **ICU Specialty, Psychiatry** | **N (%)** |  | 7 (0.04%) | 3 (0.02%) |
| **ICU Specialty, Pulmonary** | **N (%)** |  | 1270 (6.61%) | 638 (4.22%) |
| **ICU Specialty, Rehabilitation medicine** | **N (%)** |  |  | 1 (0.01%) |
| **ICU Specialty, Rheumatology** | **N (%)** |  |  | 3 (0.02%) |
| **ICU Specialty, Surgery** | **N (%)** |  | 2358 (12.27%) | 1665 (11.0%) |
| **ICU Specialty, Trauma surgery** | **N (%)** |  |  | 386 (2.55%) |
| **ICU Specialty, Unknown** | **N (%)** | 9959 (100.0%) | 3 (0.02%) |  |
| **ICU Specialty, Urology** | **N (%)** |  | 157 (0.82%) | 113 (0.75%) |
| **ICU Specialty, Vascular surgery** | **N (%)** |  |  | 471 (3.11%) |

## Supplementary Table S5. Patient characteristics, stratified per hospital. Missingness per feature is reported in Supplementary Table S6.

|  | **Missing, N (%)** | | |
| --- | --- | --- | --- |
| **Feature** | **MSZ** | **OLVG** | **AUMC** |
| **Age** | 0 (0.0%) | 0 (0.0%) | 0 (0.0%) |
| **Sex, Male** | 0 (0.0%) | 0 (0.0%) | 0 (0.0%) |
| **BMI, last 24h, mean** | 5469 (54.92%) | 5044 (26.24%) | 7597 (50.2%) |
| **GCS, last 24h, max** | 1591 (15.98%) | 14983 (77.95%) | 4675 (30.89%) |
| **GCS, last 24h, min** | 1591 (15.98%) | 14983 (77.95%) | 4675 (30.89%) |
| **RASS, last 24h, max** | 275 (2.76%) | 18 (0.09%) | 11518 (76.11%) |
| **RASS, last 24h, min** | 275 (2.76%) | 18 (0.09%) | 11518 (76.11%) |
| **Heart rate, last 24h, mean** | 13 (0.13%) | 3 (0.02%) | 108 (0.71%) |
| **ABP systolic, last 24h, mean** | 178 (1.79%) | 1135 (5.9%) | 1043 (6.89%) |
| **ABP diastolic, last 24h, mean** | 178 (1.79%) | 1136 (5.91%) | 1042 (6.89%) |
| **Respiratory rate, last 24h, mean** | 62 (0.62%) | 7 (0.04%) | 1582 (10.45%) |
| **O2 saturation, last 24h, mean** | 41 (0.41%) | 14 (0.07%) | 571 (3.77%) |
| **Tracheobronchial toilet, last 24, count** | 9103 (91.4%) | 7428 (38.65%) | 12930 (85.44%) |
| **O2 flow, last 24h, mean** | 2047 (20.55%) | 2705 (14.07%) | 1301 (8.6%) |
| **FiO2, last 24h, mean** | 8751 (87.87%) | 8135 (42.32%) | 6555 (43.31%) |
| **PEEP, last 24h, max** | 6886 (69.14%) | 3476 (18.08%) | 2587 (17.09%) |
| **TV/kg, last 24h, mean** | 6981 (70.1%) | 4939 (25.7%) | 3071 (20.29%) |
| **PaO2/FiO2 ratio, last overall** | 6529 (65.56%) | 3821 (19.88%) | 1918 (12.67%) |
| **PaCO2, last 24h, mean** | 2031 (20.39%) | 1603 (8.34%) | 1434 (9.48%) |
| **pH arterial, last overall** | 1383 (13.89%) | 1051 (5.47%) | 854 (5.64%) |
| **BE, last 24h, mean** | 1701 (17.08%) | 1367 (7.11%) | 768 (5.07%) |
| **HCO3-, arterial, last 24h, mean** | 2031 (20.39%) | 1605 (8.35%) | 1508 (9.96%) |
| **Lactate, last overall** | 3140 (31.53%) | 2414 (12.56%) | 4959 (32.77%) |
| **Glucose, last 24h, min** | 620 (6.23%) | 558 (2.9%) | 556 (3.67%) |
| **ALAT, last 24h, mean** | 1169 (11.74%) | 8398 (43.69%) | 10537 (69.62%) |
| **ASAT, last 24h, mean** | 7479 (75.1%) | 17804 (92.63%) | 10646 (70.34%) |
| **Alkaline Phosphatase, last 24h, mean** | 7457 (74.88%) | 17916 (93.21%) | 11250 (74.34%) |
| **gGT, last 24h, mean** | 810 (8.13%) | 17926 (93.26%) | 11053 (73.03%) |
| **Bilirubin, total, last 24h, mean** | 626 (6.29%) | 10250 (53.33%) | 8582 (56.71%) |
| **LDH, last 24h, mean** | 7566 (75.97%) | 7986 (41.55%) | 10961 (72.43%) |
| **Albumin, last 24h, mean** | 271 (2.72%) | 7798 (40.57%) | 1181 (7.8%) |
| **APTT, last 24h, mean** | 731 (7.34%) | 2189 (11.39%) | 2100 (13.88%) |
| **Cardiac Output, last 24h, max** | 9929 (99.7%) | 18530 (96.4%) | 10188 (67.32%) |
| **Cardiac Output, last 24h, min** | 9929 (99.7%) | 18530 (96.4%) | 10188 (67.32%) |
| **Creatinine, last overall** | 235 (2.36%) | 1315 (6.84%) | 278 (1.84%) |
| **Ureum, last 24h, mean** | 4452 (44.7%) | 8261 (42.98%) | 6882 (45.47%) |
| **Ureum/Creatinine ratio, last 24h, mean** | 4479 (44.97%) | 8276 (43.06%) | 6914 (45.69%) |
| **Urine production, last 24h, total** | 126 (1.27%) | 85 (0.44%) | 272 (1.8%) |
| **Hb, last 24h, mean** | 113 (1.13%) | 788 (4.1%) | 245 (1.62%) |
| **Leukocytes, last 24h, mean** | 239 (2.4%) | 3748 (19.5%) | 304 (2.01%) |
| **Thrombocytes, last 24h, mean** | 486 (4.88%) | 1900 (9.89%) | 759 (5.02%) |
| **CRP, last 24h, mean** | 731 (7.34%) | 8523 (44.34%) | 7686 (50.79%) |
| **Sodium, last 24h, mean** | 557 (5.59%) | 858 (4.46%) | 537 (3.55%) |
| **Potassium, last 24h, mean** | 512 (5.14%) | 862 (4.48%) | 530 (3.5%) |
| **Chloride, last 24h, mean** | 1292 (12.97%) | 1328 (6.91%) | 3112 (20.56%) |
| **Calcium, ionized, last 24h, mean** | 1833 (18.41%) | 3187 (16.58%) | 3344 (22.1%) |
| **Phosphate, last 24h, mean** | 607 (6.09%) | 1935 (10.07%) | 1481 (9.79%) |
| **Magnesium,last 24h, mean** | 579 (5.81%) | 1916 (9.97%) | 2000 (13.22%) |

## Supplementary Table S6. Continuation of Supplementary Table 5 Missingness per feature. Calculated features and outcomes for which no missingness could be reported, such as length of stay and categorical subgroups are removed from this table.

|  |  | **Missing, N (%)** | | **Value** | |
| --- | --- | --- | --- | --- | --- |
| **Feature** | **Description** | **OLVG** | **AUMC** | **OLVG** | **AUMC** |
| **Number of patients** | **N (%)** |  |  | 11512 (100.0%) | 7179 (100.0%) |
| **Age** | **Median [IQR]** |  |  | 68.0 [61.0, 75.0] | 69.36 [61.85, 76.0] |
| **Age (<50)** | **N (%)** |  |  | 688 (5.98%) | 405 (5.64%) |
| **Age (50-70)** | **N (%)** |  |  | 5702 (49.53%) | 3362 (46.83%) |
| **Age (70-80)** | **N (%)** |  |  | 4155 (36.09%) | 2669 (37.18%) |
| **Age (>80)** | **N (%)** |  |  | 967 (8.4%) | 743 (10.35%) |
| **Sex, Male** | **N (%)** |  |  | 8135 (70.67%) | 5094 (70.96%) |
| **BMI, last 24h, mean** | **Median [IQR]** | 2995 (26.02%) | 2677 (37.29%) | 26.87 [24.4, 29.76] | 26.4 [24.38, 29.05] |
| **Length of Stay** | **Median [IQR]** |  |  | 22.25 [19.08, 42.12] | 22.83 [20.47, 25.25] |
| **Length of Stay (4-12 hours)** | **N (%)** |  | | 1 (0.01%) |  |
| **Length of Stay (12-24 hours)** | **N (%)** |  |  | 7163 (62.22%) | 4685 (65.26%) |
| **Length of Stay (1-3 days)** | **N (%)** |  |  | 3173 (27.56%) | 1969 (27.43%) |
| **Length of Stay (3-7 days)** | **N (%)** |  |  | 830 (7.21%) | 323 (4.5%) |
| **Length of Stay (7-14 days)** | **N (%)** |  |  | 231 (2.01%) | 119 (1.66%) |
| **Length of Stay (>14 days)** | **N (%)** |  |  | 114 (0.99%) | 83 (1.16%) |
| **Readmission or Mortality after discharge, 7d** | **N (%)** |  |  | 492 (4.27%) | 158 (2.2%) |
| **Readmission after discharge, 7d** | **N (%)** |  |  | 475 (4.13%) | 145 (2.02%) |
| **GCS, last 24h, max** | **Median [IQR]** | 8917 (77.46%) | 2202 (30.67%) | 3.0 [3.0, 14.0] | 15.0 [15.0, 15.0] |
| **GCS, last 24h, min** | **Median [IQR]** | 8917 (77.46%) | 2202 (30.67%) | 3.0 [3.0, 6.0] | 15.0 [15.0, 15.0] |
| **RASS, last 24h, max** | **Median [IQR]** | 4 (0.03%) | 5837 (81.31%) | 0.0 [0.0, 0.0] | 0.0 [0.0, 0.0] |
| **RASS, last 24h, min** | **Median [IQR]** | 4 (0.03%) | 5837 (81.31%) | -3.0 [-4.0, -1.0] | 0.0 [-3.0, 0.0] |
| **Heart rate, last 24h, mean** | **Median [IQR]** | 0 (0.0%) | 45 (0.63%) | 78.57 [71.53, 85.87] | 78.41 [71.22, 85.7] |
| **ABP systolic, last 24h, mean** | **Median [IQR]** | 328 (2.85%) | 83 (1.16%) | 117.21 [107.75, 129.01] | 124.5 [113.71, 135.1] |
| **ABP diastolic, last 24h, mean** | **Median [IQR]** | 330 (2.87%) | 83 (1.16%) | 58.46 [53.71, 63.96] | 60.36 [55.48, 65.95] |
| **Respiratory rate, last 24h, mean** | **Median [IQR]** | 1 (0.01%) | 243 (3.38%) | 15.46 [13.48, 18.0] | 15.45 [14.0, 17.45] |
| **O2 saturation, last 24h, mean** | **Median [IQR]** | 2 (0.02%) | 120 (1.67%) | 96.38 [95.27, 97.5] | 96.5 [94.0, 98.0] |
| **Tracheobronchial toilet, last 24, count** | **Median [IQR]** | 4137 (35.94%) | 6247 (87.02%) | 1.0 [1.0, 2.0] | 2.0 [1.0, 2.0] |
| **O2 flow, last 24h, mean** | **Median [IQR]** | 1008 (8.76%) | 113 (1.57%) | 2.25 [1.6, 3.29] | 5.0 [3.4, 5.4] |
| **FiO2, last 24h, mean** | **Median [IQR]** | 3449 (29.96%) | 1206 (16.8%) | 33.57 [27.0, 40.0] | 40.54 [40.03, 45.08] |
| **PEEP, last 24h, max** | **Median [IQR]** | 730 (6.34%) | 136 (1.89%) | 8.16 [8.16, 8.2] | 6.0 [5.2, 8.2] |
| **TV/kg, last 24h, mean** | **Median [IQR]** | 1668 (14.49%) | 239 (3.33%) | 7.62 [6.83, 8.46] | 7.2 [6.51, 8.0] |
| **PaO2/FiO2 ratio, last overall** | **Median [IQR]** | 1244 (10.81%) | 287 (4.0%) | 260.0 [197.14, 333.33] | 255.53 [205.0, 328.57] |
| **PaCO2, last 24h, mean** | **Median [IQR]** | 722 (6.27%) | 269 (3.75%) | 40.25 [37.5, 43.0] | 39.0 [36.66, 41.5] |
| **pH arterial, last overall** | **Median [IQR]** | 518 (4.5%) | 263 (3.66%) | 7.42 [7.4, 7.45] | 7.41 [7.38, 7.44] |
| **BE, last 24h, mean** | **Median [IQR]** | 630 (5.47%) | 85 (1.18%) | -0.05 [-1.4, 1.53] | 0.99 [-1.31, 2.16] |
| **HCO3-, arterial, last 24h, mean** | **Median [IQR]** | 724 (6.29%) | 284 (3.96%) | 24.32 [23.05, 25.78] | 22.92 [21.77, 24.13] |
| **Lactate, last overall** | **Median [IQR]** | 1804 (15.67%) | 3665 (51.05%) | 1.3 [1.0, 1.9] | 1.4 [1.1, 1.9] |
| **Glucose, last 24h, min** | **Median [IQR]** | 207 (1.8%) | 74 (1.03%) | 6.9 [5.9, 7.9] | 6.8 [5.7, 7.8] |
| **ALAT, last 24h, mean** | **Median [IQR]** | 7221 (62.73%) | 6120 (85.25%) | 29.0 [20.0, 47.0] | 24.0 [17.0, 37.0] |
| **ASAT, last 24h, mean** | **Median [IQR]** | 11264 (97.85%) | 6141 (85.54%) | 48.0 [34.0, 76.0] | 46.0 [32.0, 68.0] |
| **Alkaline Phosphatase, last 24h, mean** | **Median [IQR]** | 11410 (99.11%) | 6447 (89.8%) | 69.0 [51.25, 110.62] | 54.0 [43.0, 74.0] |
| **gGT, last 24h, mean** | **Median [IQR]** | 11412 (99.13%) | 6427 (89.53%) | 52.0 [22.75, 119.0] | 29.0 [18.0, 58.0] |
| **Bilirubin, total, last 24h, mean** | **Median [IQR]** | 7987 (69.38%) | 5355 (74.59%) | 9.0 [6.0, 12.0] | 8.0 [6.0, 11.0] |
| **LDH, last 24h, mean** | **Median [IQR]** | 7140 (62.02%) | 6203 (86.4%) | 282.0 [218.0, 363.0] | 315.0 [247.0, 414.17] |
| **Albumin, last 24h, mean** | **Median [IQR]** | 7073 (61.44%) | 655 (9.12%) | 31.0 [29.0, 34.0] | 25.0 [22.0, 27.5] |
| **APTT, last 24h, mean** | **Median [IQR]** | 1007 (8.75%) | 262 (3.65%) | 26.0 [24.0, 28.5] | 37.75 [34.75, 41.67] |
| **Cardiac Output, last 24h, is measured** | **N (%)** |  |  | 384 (3.34%) | 4706 (65.55%) |
| **Cardiac Output, last 24h, max** | **Median [IQR]** | 11128 (96.66%) | 2473 (34.45%) | 6.7 [5.8, 8.0] | 5.9 [5.0, 6.9] |
| **Cardiac Output, last 24h, min** | **Median [IQR]** | 11128 (96.66%) | 2473 (34.45%) | 4.6 [3.88, 5.84] | 4.5 [3.7, 5.3] |
| **CK-MB, last 24h, is measured** | **N (%)** |  |  | 8736 (75.89%) | 6236 (86.86%) |
| **Creatinine, last overall** | **Median [IQR]** | 739 (6.42%) | 61 (0.85%) | 77.0 [64.0, 94.0] | 82.0 [68.0, 99.0] |
| **Ureum, last 24h, mean** | **Median [IQR]** | 7157 (62.17%) | 4543 (63.28%) | 8.7 [6.5, 12.2] | 6.8 [5.55, 8.75] |
| **Ureum/Creatinine ratio, last 24h, mean** | **Median [IQR]** | 7160 (62.2%) | 4549 (63.37%) | 0.11 [0.08, 0.14] | 0.08 [0.07, 0.1] |
| **Urine production, last 24h, total** | **Median [IQR]** | 7 (0.06%) | 65 (0.91%) | 1.83 [1.34, 2.50] | 1.70 [1.28, 2.26] |
| **Hb, last 24h, mean** | **Median [IQR]** | 428 (3.72%) | 60 (0.84%) | 6.4 [5.77, 7.18] | 6.75 [6.25, 7.37] |
| **Leukocytes, last 24h, mean** | **Median [IQR]** | 3077 (26.73%) | 60 (0.84%) | 15.5 [12.0, 18.6] | 13.8 [11.4, 16.55] |
| **Thrombocytes, last 24h, mean** | **Median [IQR]** | 932 (8.1%) | 95 (1.32%) | 176.0 [141.0, 221.0] | 160.0 [128.0, 199.5] |
| **CRP, last 24h, mean** | **Median [IQR]** | 7339 (63.75%) | 4955 (69.02%) | 49.0 [31.0, 78.0] | 30.0 [18.0, 53.0] |
| **Sodium, last 24h, mean** | **Median [IQR]** | 360 (3.13%) | 75 (1.04%) | 138.0 [136.33, 139.5] | 138.75 [137.0, 140.6] |
| **Potassium, last 24h, mean** | **Median [IQR]** | 361 (3.14%) | 75 (1.04%) | 4.3 [4.06, 4.54] | 4.23 [4.03, 4.47] |
| **Chloride, last 24h, mean** | **Median [IQR]** | 853 (7.41%) | 1642 (22.87%) | 107.2 [105.0, 109.0] | 107.89 [105.5, 110.14] |
| **Calcium, ionized, last 24h, mean** | **Median [IQR]** | 1908 (16.57%) | 1685 (23.47%) | 1.15 [1.12, 1.19] | 1.17 [1.14, 1.2] |
| **Phosphate, last 24h, mean** | **Median [IQR]** | 965 (8.38%) | 588 (8.19%) | 1.06 [0.9, 1.23] | 0.99 [0.82, 1.17] |
| **Magnesium,last 24h, mean** | **Median [IQR]** | 930 (8.08%) | 492 (6.85%) | 1.02 [0.87, 1.21] | 1.04 [0.93, 1.15] |

## Supplementary Table S7. Patient characteristics of cardiac surgery patients for hospitals OLVG and AUMC.

| **Feature** | **description** | **MSZ** | **OLVG** | **AUMC** |
| --- | --- | --- | --- | --- |
| **Patients** | **N (%)** | 9959 (100.0%) | 7709 (100.0%) | 7955 (100.0%) |
| **Age** | **Median [IQR]** | 65.0 [54.0, 74.0] | 65.0 [54.0, 74.0] | 62.71 [48.13, 73.11] |
| **Age, <50** | **N (%)** | 1932 (19.4%) | 1455 (18.87%) | 2172 (27.3%) |
| **Age, 50-70** | **N (%)** | 4610 (46.29%) | 3412 (44.26%) | 3248 (40.83%) |
| **Age, 70-80** | **N (%)** | 2547 (25.57%) | 1960 (25.42%) | 1785 (22.44%) |
| **Age, >80** | **N (%)** | 870 (8.74%) | 882 (11.44%) | 750 (9.43%) |
| **Sex, Male** | **N (%)** | 5987 (60.12%) | 4851 (62.93%) | 5017 (63.07%) |
| **BMI, last 24h, mean** | **Median [IQR]** | 26.42 [23.53, 30.86] | 25.88 [22.82, 29.58] | 24.57 [22.38, 27.34] |
| **Length of Stay** | **Median [IQR]** | 41.27 [21.3, 85.7] | 45.2 [22.0, 98.87] | 45.67 [22.75, 114.58] |
| **Length of Stay, 4-12 hours** | **N (%)** |  | 6 (0.08%) | 5 (0.06%) |
| **Length of Stay, 12-24 hours** | **N (%)** | 3436 (34.5%) | 2184 (28.33%) | 2299 (28.9%) |
| **Length of Stay, 1-3 days** | **N (%)** | 3727 (37.42%) | 2909 (37.74%) | 2777 (34.91%) |
| **Length of Stay, 3-7 days** | **N (%)** | 1700 (17.07%) | 1575 (20.43%) | 1565 (19.67%) |
| **Length of Stay, 7-14 days** | **N (%)** | 723 (7.26%) | 708 (9.18%) | 828 (10.41%) |
| **Length of Stay, >14 days** | **N (%)** | 373 (3.75%) | 327 (4.24%) | 481 (6.05%) |
| **Readmission/Mortality after discharge, 7d** | **N (%)** | 706 (7.09%) | 829 (10.75%) | 733 (9.21%) |
| **Readmission after discharge, 7d** | **N (%)** | 577 (5.79%) | 678 (8.79%) | 577 (7.25%) |
| **Mortality, 7d** | **N (%)** | 175 (1.76%) | 191 (2.48%) | 199 (2.5%) |
| **Mortality, 30d** | **N (%)** | 476 (4.78%) | 436 (5.66%) | 436 (5.48%) |
| **Mortality, 90d** | **N (%)** | 775 (7.78%) | 532 (6.9%) | 591 (7.43%) |
| **GCS, last 24h, max** | **Median [IQR]** | 15.0 [15.0, 15.0] | 14.0 [9.0, 15.0] | 15.0 [15.0, 15.0] |
| **GCS, last 24h, min** | **Median [IQR]** | 15.0 [14.0, 15.0] | 10.0 [3.0, 14.0] | 15.0 [13.0, 15.0] |
| **RASS, last 24h, max** | **Median [IQR]** | 0.0 [0.0, 0.0] | 0.0 [0.0, 1.0] | 0.0 [0.0, 0.0] |
| **RASS, last 24h, min** | **Median [IQR]** | -1.0 [-1.0, 0.0] | -1.0 [-2.0, 0.0] | 0.0 [-1.0, 0.0] |
| **Heart rate, last 24h, mean** | **Median [IQR]** | 81.23 [71.28, 91.75] | 86.3 [76.55, 97.0] | 83.55 [73.33, 94.31] |
| **ABP systolic, last 24h, mean** | **Median [IQR]** | 127.33 [114.55, 142.15] | 122.13 [110.17, 137.39] | 128.05 [114.72, 143.45] |
| **ABP diastolic, last 24h, mean** | **Median [IQR]** | 63.11 [55.7, 72.14] | 60.67 [54.1, 68.22] | 63.35 [57.05, 71.14] |
| **Respiratory rate, last 24h, mean** | **Median [IQR]** | 18.17 [15.9, 20.91] | 19.08 [16.43, 22.13] | 18.23 [15.67, 21.49] |
| **O2 saturation, last 24h, mean** | **Median [IQR]** | 97.04 [95.78, 98.16] | 96.07 [94.75, 97.46] | 96.83 [95.37, 98.0] |
| **Tracheobronchial toilet, last 24, count** | **Median [IQR]** | 2.0 [1.0, 3.0] | 0.0 [0.0, 2.0] | 3.0 [1.0, 5.0] |
| **O2 flow, last 24h, mean** | **Median [IQR]** | 2.0 [1.55, 3.82] | 2.5 [1.71, 3.71] | 3.86 [2.5, 5.0] |
| **FiO2, last 24h, mean** | **Median [IQR]** | 40.0 [32.99, 46.67] | 36.17 [28.17, 41.0] | 40.25 [38.46, 45.03] |
| **PEEP, last 24h, max** | **Median [IQR]** | 10.0 [7.8, 12.1] | 8.16 [8.16, 11.22] | 8.0 [5.3, 10.4] |
| **TV/kg, last 24h, mean** | **Median [IQR]** | 7.68 [6.91, 8.61] | 7.36 [6.34, 8.51] | 7.14 [6.36, 8.1] |
| **PaO2/FiO2 ratio, last overall** | **Median [IQR]** | 235.0 [183.62, 308.57] | 240.0 [182.86, 320.66] | 285.0 [210.0, 370.0] |
| **PaCO2, last 24h, mean** | **Median [IQR]** | 38.17 [34.5, 42.29] | 38.0 [33.75, 43.0] | 38.83 [35.25, 42.76] |
| **pH arterial, last overall** | **Median [IQR]** | 7.43 [7.4, 7.46] | 7.44 [7.4, 7.47] | 7.43 [7.4, 7.46] |
| **BE, last 24h, mean** | **Median [IQR]** | 0.5 [-1.67, 2.83] | 0.95 [-1.9, 3.88] | 1.72 [0.1, 3.6] |
| **HCO3-, arterial, last 24h, mean** | **Median [IQR]** | 24.71 [22.5, 27.0] | 24.7 [21.98, 27.75] | 24.56 [22.4, 27.07] |
| **Lactate, last overall** | **Median [IQR]** | 1.3 [0.9, 1.8] | 1.2 [0.9, 1.6] | 1.1 [0.8, 1.6] |
| **Glucose, last 24h, min** | **Median [IQR]** | 6.1 [5.3, 7.1] | 6.2 [5.4, 7.2] | 6.1 [5.3, 7.0] |
| **ALAT, last 24h, mean** | **Median [IQR]** | 27.0 [16.0, 54.0] | 35.0 [20.0, 71.0] | 32.0 [18.0, 67.0] |
| **ASAT, last 24h, mean** | **Median [IQR]** | 31.0 [23.0, 56.0] | 39.0 [23.0, 85.0] | 37.5 [23.0, 68.0] |
| **Alkaline Phosphatase, last 24h, mean** | **Median [IQR]** | 73.0 [56.0, 99.0] | 87.0 [62.0, 129.0] | 77.0 [57.0, 115.0] |
| **gGT, last 24h, mean** | **Median [IQR]** | 41.0 [21.0, 99.0] | 54.0 [24.0, 128.0] | 45.0 [21.0, 112.0] |
| **Bilirubin, total, last 24h, mean** | **Median [IQR]** | 8.5 [6.0, 12.0] | 8.0 [5.0, 12.0] | 8.0 [6.0, 12.54] |
| **LDH, last 24h, mean** | **Median [IQR]** | 222.0 [182.0, 306.0] | 234.0 [179.0, 327.0] | 245.0 [186.0, 347.0] |
| **Albumin, last 24h, mean** | **Median [IQR]** | 25.0 [21.0, 30.0] | 29.0 [25.0, 33.0] | 24.0 [20.0, 28.0] |
| **APTT, last 24h, mean** | **Median [IQR]** | 29.0 [26.0, 33.0] | 25.85 [23.0, 30.05] | 38.0 [32.5, 45.5] |
| **Cardiac Output, last 24h, is measured** | **N (%)** | 30 (0.3%) | 307 (3.98%) | 240 (3.02%) |
| **Cardiac Output, last 24h, max** | **Median [IQR]** | 6.78 [5.54, 8.81] | 8.11 [6.75, 10.0] | 6.3 [5.2, 7.52] |
| **Cardiac Output, last 24h, min** | **Median [IQR]** | 5.3 [3.85, 6.16] | 5.0 [4.1, 6.1] | 4.6 [3.9, 5.7] |
| **CK-MB, last 24h, is measured** | **N (%)** | 710 (7.13%) | 2203 (28.58%) | 1774 (22.3%) |
| **Creatinine, last overall** | **Median [IQR]** | 69.0 [52.0, 98.0] | 77.0 [58.0, 121.0] | 70.0 [54.0, 96.0] |
| **Ureum, last 24h, mean** | **Median [IQR]** | 6.55 [4.7, 10.0] | 9.2 [6.0, 14.6] | 6.5 [4.35, 10.0] |
| **Ureum/Creatinine ratio, last 24h, mean** | **Median [IQR]** | 0.09 [0.06, 0.12] | 0.11 [0.08, 0.15] | 0.08 [0.06, 0.12] |
| **Urine production, last 24h, total** | **Median [IQR]** | 1458.34 [967.6, 2198.33] | 2004.0 [1284.5, 2957.29] | 1671.46 [1108.94, 2483.29] |
| **Hb, last 24h, mean** | **Median [IQR]** | 6.8 [6.0, 7.75] | 6.45 [5.68, 7.5] | 6.69 [5.88, 7.6] |
| **Leukocytes, last 24h, mean** | **Median [IQR]** | 11.1 [8.6, 14.4] | 12.3 [9.0, 16.6] | 11.1 [8.47, 14.4] |
| **Thrombocytes, last 24h, mean** | **Median [IQR]** | 225.0 [168.0, 307.0] | 214.5 [154.0, 294.0] | 209.0 [150.0, 301.0] |
| **CRP, last 24h, mean** | **Median [IQR]** | 63.0 [24.08, 131.0] | 48.0 [17.0, 109.0] | 61.0 [23.0, 127.0] |
| **Sodium, last 24h, mean** | **Median [IQR]** | 138.0 [135.67, 140.5] | 139.0 [136.5, 142.0] | 138.5 [136.0, 141.0] |
| **Potassium, last 24h, mean** | **Median [IQR]** | 4.0 [3.8, 4.23] | 4.04 [3.85, 4.28] | 4.0 [3.8, 4.25] |
| **Chloride, last 24h, mean** | **Median [IQR]** | 105.75 [103.0, 108.5] | 106.8 [103.5, 110.0] | 106.0 [102.67, 109.5] |
| **Calcium, ionized, last 24h, mean** | **Median [IQR]** | 1.13 [1.1, 1.17] | 1.19 [1.14, 1.24] | 1.17 [1.13, 1.21] |
| **Phosphate, last 24h, mean** | **Median [IQR]** | 1.07 [0.89, 1.27] | 1.06 [0.87, 1.28] | 1.02 [0.83, 1.23] |
| **Magnesium,last 24h, mean** | **Median [IQR]** | 0.86 [0.78, 0.97] | 0.86 [0.76, 0.98] | 0.82 [0.74, 0.9] |
| **ICU Specialty, Anesthesiology** | **N (%)** |  |  | 2 (0.03%) |
| **ICU Specialty, Cardiology** | **N (%)** |  | 1222 (15.85%) | 961 (12.08%) |
| **ICU Specialty, ENT** | **N (%)** |  | 48 (0.62%) | 308 (3.87%) |
| **ICU Specialty, Gastroenterology** | **N (%)** |  | 357 (4.63%) | 167 (2.1%) |
| **ICU Specialty, Gynecology** | **N (%)** |  | 87 (1.13%) | 134 (1.68%) |
| **ICU Specialty, Hematology** | **N (%)** |  | 36 (0.47%) |  |
| **ICU Specialty, Intensive care** | **N (%)** |  |  | 632 (7.94%) |
| **ICU Specialty, Internal medicine** | **N (%)** |  | 1746 (22.65%) | 1152 (14.48%) |
| **ICU Specialty, MKA** | **N (%)** |  | 28 (0.36%) | 28 (0.35%) |
| **ICU Specialty, Neuro surgery** | **N (%)** |  |  | 557 (7.0%) |
| **ICU Specialty, Neurology** | **N (%)** |  | 230 (2.98%) | 542 (6.81%) |
| **ICU Specialty, Oncology** | **N (%)** |  | 12 (0.16%) | 32 (0.4%) |
| **ICU Specialty, Ophthalmology** | **N (%)** |  |  | 5 (0.06%) |
| **ICU Specialty, Orthopedic surgery** | **N (%)** |  | 136 (1.76%) | 106 (1.33%) |
| **ICU Specialty, Other** | **N (%)** |  | 5 (0.06%) | 27 (0.34%) |
| **ICU Specialty, Plastic surgery** | **N (%)** |  | 7 (0.09%) | 22 (0.28%) |
| **ICU Specialty, Psychiatry** | **N (%)** |  | 7 (0.09%) | 3 (0.04%) |
| **ICU Specialty, Pulmonary** | **N (%)** |  | 1270 (16.47%) | 638 (8.02%) |
| **ICU Specialty, Rehabilitation medicine** | **N (%)** |  |  | 1 (0.01%) |
| **ICU Specialty, Rheumatology** | **N (%)** |  |  | 3 (0.04%) |
| **ICU Specialty, Surgery** | **N (%)** |  | 2358 (30.59%) | 1665 (20.93%) |
| **ICU Specialty, Trauma surgery** | **N (%)** |  |  | 386 (4.85%) |
| **ICU Specialty, Unknown** | **N (%)** | 9959 (100.0%) | 3 (0.04%) |  |
| **ICU Specialty, Urology** | **N (%)** |  | 157 (2.04%) | 113 (1.42%) |
| **ICU Specialty, Vascular surgery** | **N (%)** |  |  | 471 (5.92%) |

## Supplementary Table S8. Patient characteristics of all hospitals after removal of cardiac surgery patients.

| **Feature** | **MSZ** | **OLVG** | **AUMC** |
| --- | --- | --- | --- |
| **Age** | 0 (0.0%) | 0 (0.0%) | 0 (0.0%) |
| **Sex, Male** | 0 (0.0%) | 0 (0.0%) | 0 (0.0%) |
| **BMI, last 24h, mean** | 5469 (54.92%) | 2049 (26.58%) | 4920 (61.85%) |
| **GCS, last 24h, max** | 1591 (15.98%) | 6066 (78.69%) | 2473 (31.09%) |
| **GCS, last 24h, min** | 1591 (15.98%) | 6066 (78.69%) | 2473 (31.09%) |
| **RASS, last 24h, max** | 275 (2.76%) | 14 (0.18%) | 5681 (71.41%) |
| **RASS, last 24h, min** | 275 (2.76%) | 14 (0.18%) | 5681 (71.41%) |
| **Heart rate, last 24h, mean** | 13 (0.13%) | 3 (0.04%) | 63 (0.79%) |
| **ABP systolic, last 24h, mean** | 178 (1.79%) | 807 (10.47%) | 960 (12.07%) |
| **ABP diastolic, last 24h, mean** | 178 (1.79%) | 806 (10.46%) | 959 (12.06%) |
| **Respiratory rate, last 24h, mean** | 62 (0.62%) | 6 (0.08%) | 1339 (16.83%) |
| **O2 saturation, last 24h, mean** | 41 (0.41%) | 12 (0.16%) | 451 (5.67%) |
| **Tracheobronchial toilet, last 24, count** | 9103 (91.4%) | 3291 (42.69%) | 6683 (84.01%) |
| **O2 flow, last 24h, mean** | 2047 (20.55%) | 1697 (22.01%) | 1188 (14.93%) |
| **FiO2, last 24h, mean** | 8751 (87.87%) | 4686 (60.79%) | 5349 (67.24%) |
| **PEEP, last 24h, max** | 6886 (69.14%) | 2746 (35.62%) | 2451 (30.81%) |
| **TV/kg, last 24h, mean** | 6981 (70.1%) | 3271 (42.43%) | 2832 (35.6%) |
| **PaO2/FiO2 ratio, last overall** | 6529 (65.56%) | 2577 (33.43%) | 1631 (20.5%) |
| **PaCO2, last 24h, mean** | 2031 (20.39%) | 881 (11.43%) | 1165 (14.64%) |
| **pH arterial, last overall** | 1383 (13.89%) | 533 (6.91%) | 591 (7.43%) |
| **BE, last 24h, mean** | 1701 (17.08%) | 737 (9.56%) | 683 (8.59%) |
| **HCO3-, arterial, last 24h, mean** | 2031 (20.39%) | 881 (11.43%) | 1224 (15.39%) |
| **Lactate, last overall** | 3140 (31.53%) | 610 (7.91%) | 1294 (16.27%) |
| **Glucose, last 24h, min** | 620 (6.23%) | 351 (4.55%) | 482 (6.06%) |
| **ALAT, last 24h, mean** | 1169 (11.74%) | 1177 (15.27%) | 4417 (55.52%) |
| **ASAT, last 24h, mean** | 7479 (75.1%) | 6540 (84.84%) | 4505 (56.63%) |
| **Alkaline Phosphatase, last 24h, mean** | 7457 (74.88%) | 6506 (84.39%) | 4803 (60.38%) |
| **gGT, last 24h, mean** | 810 (8.13%) | 6514 (84.5%) | 4626 (58.15%) |
| **Bilirubin, total, last 24h, mean** | 626 (6.29%) | 2263 (29.36%) | 3227 (40.57%) |
| **LDH, last 24h, mean** | 7566 (75.97%) | 846 (10.97%) | 4758 (59.81%) |
| **Albumin, last 24h, mean** | 271 (2.72%) | 725 (9.4%) | 526 (6.61%) |
| **APTT, last 24h, mean** | 731 (7.34%) | 1182 (15.33%) | 1838 (23.1%) |
| **Cardiac Output, last 24h, max** | 9929 (99.7%) | 7402 (96.02%) | 7715 (96.98%) |
| **Cardiac Output, last 24h, min** | 9929 (99.7%) | 7402 (96.02%) | 7715 (96.98%) |
| **Creatinine, last overall** | 235 (2.36%) | 576 (7.47%) | 217 (2.73%) |
| **Ureum, last 24h, mean** | 4452 (44.7%) | 1104 (14.32%) | 2339 (29.4%) |
| **Ureum/Creatinine ratio, last 24h, mean** | 4479 (44.97%) | 1116 (14.48%) | 2365 (29.73%) |
| **Urine production, last 24h, total** | 126 (1.27%) | 78 (1.01%) | 207 (2.6%) |
| **Hb, last 24h, mean** | 113 (1.13%) | 360 (4.67%) | 185 (2.33%) |
| **Leukocytes, last 24h, mean** | 239 (2.4%) | 671 (8.7%) | 244 (3.07%) |
| **Thrombocytes, last 24h, mean** | 486 (4.88%) | 968 (12.56%) | 664 (8.35%) |
| **CRP, last 24h, mean** | 731 (7.34%) | 1184 (15.36%) | 2731 (34.33%) |
| **Sodium, last 24h, mean** | 557 (5.59%) | 498 (6.46%) | 462 (5.81%) |
| **Potassium, last 24h, mean** | 512 (5.14%) | 501 (6.5%) | 455 (5.72%) |
| **Chloride, last 24h, mean** | 1292 (12.97%) | 475 (6.16%) | 1470 (18.48%) |
| **Calcium, ionized, last 24h, mean** | 1833 (18.41%) | 1279 (16.59%) | 1659 (20.85%) |
| **Phosphate, last 24h, mean** | 607 (6.09%) | 970 (12.58%) | 893 (11.23%) |
| **Magnesium,last 24h, mean** | 579 (5.81%) | 986 (12.79%) | 1508 (18.96%) |

## Supplementary Table S9. Data availability of features in all hospitals after removal of cardiac surgery patients, corresponding to Supplementary Table S8.

|  |  |  |  |  |  |  | **CTC** |  |  |  |
| --- | --- | --- | --- | --- | --- | --- | --- | --- | --- | --- |
| **dataset** | **model** | **fold** | **AUROC** | **95%CI** | **positive** | **size** | **AUROC** | **95%CI** | **positive** | **size** |
| msz | msz | 0 | 69.7 | (64.8, 74.7) | 141 | 1992 |  |  |  |  |
|  |  | 1 | 72.6 | (67.8, 77.5) | 141 | 1992 |  |  |  |  |
|  |  | 2 | 70.4 | (65.5, 75.4) | 141 | 1992 |  |  |  |  |
|  |  | 3 | 69.1 | (64.1, 74.1) | 142 | 1992 |  |  |  |  |
|  |  | 4 | 72.4 | (67.5, 77.3) | 141 | 1991 |  |  |  |  |
|  | olvg | 0 | 68.1 | (63.1, 73.1) | 141 | 1992 |  |  |  |  |
|  |  | 1 | 71.0 | (66.1, 75.9) | 141 | 1992 |  |  |  |  |
|  |  | 2 | 70.1 | (65.2, 75.1) | 141 | 1992 |  |  |  |  |
|  |  | 3 | 70.1 | (65.1, 75.0) | 142 | 1992 |  |  |  |  |
|  |  | 4 | 73.4 | (68.6, 78.3) | 141 | 1991 |  |  |  |  |
|  | pooled | 0 | 68.6 | (63.6, 73.6) | 141 | 1992 |  |  |  |  |
|  |  | 1 | 71.4 | (66.5, 76.3) | 141 | 1992 |  |  |  |  |
|  |  | 2 | 70.7 | (65.8, 75.6) | 141 | 1992 |  |  |  |  |
|  |  | 3 | 70.1 | (65.2, 75.0) | 142 | 1992 |  |  |  |  |
|  |  | 4 | 73.4 | (68.6, 78.3) | 141 | 1991 |  |  |  |  |
|  | aumc | 0 | 67.1 | (62.0, 72.1) | 141 | 1992 |  |  |  |  |
|  |  | 1 | 69.2 | (64.2, 74.2) | 141 | 1992 |  |  |  |  |
|  |  | 2 | 68.7 | (63.7, 73.7) | 141 | 1992 |  |  |  |  |
|  |  | 3 | 70.1 | (65.2, 75.0) | 142 | 1992 |  |  |  |  |
|  |  | 4 | 70.9 | (66.0, 75.8) | 141 | 1991 |  |  |  |  |
| olvg | msz | 0 | 66.1 | (62.5, 69.8) | 265 | 3845 | 62.5 | (56.6, 68.3) | 104 | 2273 |
|  |  | 1 | 66.8 | (63.2, 70.5) | 264 | 3844 | 59.4 | (53.3, 65.5) | 95 | 2291 |
|  |  | 2 | 73.6 | (70.1, 77.2) | 264 | 3844 | 63.8 | (57.8, 69.9) | 98 | 2296 |
|  |  | 3 | 69.3 | (65.6, 72.9) | 264 | 3844 | 66.3 | (60.5, 72.1) | 104 | 2334 |
|  |  | 4 | 68.8 | (65.2, 72.5) | 264 | 3844 | 68.4 | (62.2, 74.5) | 91 | 2318 |
|  | olvg | 0 | 71.2 | (67.6, 74.8) | 265 | 3845 | 70.7 | (65.0, 76.4) | 104 | 2273 |
|  |  | 1 | 75.5 | (72.0, 79.0) | 264 | 3844 | 73.0 | (67.1, 78.8) | 95 | 2291 |
|  |  | 2 | 73.6 | (70.0, 77.1) | 264 | 3844 | 66.9 | (60.9, 72.8) | 98 | 2296 |
|  |  | 3 | 74.6 | (71.1, 78.1) | 264 | 3844 | 72.5 | (66.8, 78.1) | 104 | 2334 |
|  |  | 4 | 74.9 | (71.5, 78.4) | 264 | 3844 | 75.2 | (69.3, 81.0) | 91 | 2318 |
|  | pooled | 0 | 71.3 | (67.7, 74.9) | 265 | 3845 | 69.9 | (64.1, 75.6) | 104 | 2273 |
|  |  | 1 | 75.4 | (71.9, 78.8) | 264 | 3844 | 73.3 | (67.4, 79.1) | 95 | 2291 |
|  |  | 2 | 74.2 | (70.7, 77.7) | 264 | 3844 | 67.0 | (61.0, 73.0) | 98 | 2296 |
|  |  | 3 | 74.6 | (71.1, 78.0) | 264 | 3844 | 72.3 | (66.6, 77.9) | 104 | 2334 |
|  |  | 4 | 74.7 | (71.2, 78.2) | 264 | 3844 | 74.8 | (68.9, 80.7) | 91 | 2318 |
|  | aumc | 0 | 67.7 | (64.0, 71.4) | 265 | 3845 | 61.8 | (55.9, 67.6) | 104 | 2273 |
|  |  | 1 | 72.8 | (69.3, 76.4) | 264 | 3844 | 69.5 | (63.5, 75.5) | 95 | 2291 |
|  |  | 2 | 70.8 | (67.2, 74.4) | 264 | 3844 | 61.8 | (55.8, 67.9) | 98 | 2296 |
|  |  | 3 | 71.0 | (67.4, 74.6) | 264 | 3844 | 68.0 | (62.2, 73.8) | 104 | 2334 |
|  |  | 4 | 72.6 | (69.0, 76.1) | 264 | 3844 | 68.3 | (62.1, 74.5) | 91 | 2318 |
| pooled | msz | 0 | 67.3 | (64.3, 70.2) | 406 | 5837 |  |  |  |  |
|  |  | 1 | 69.0 | (66.0, 71.9) | 405 | 5836 |  |  |  |  |
|  |  | 2 | 72.2 | (69.3, 75.0) | 405 | 5836 |  |  |  |  |
|  |  | 3 | 69.0 | (66.1, 72.0) | 406 | 5836 |  |  |  |  |
|  |  | 4 | 70.2 | (67.2, 73.1) | 405 | 5835 |  |  |  |  |
|  | olvg | 0 | 69.5 | (66.6, 72.4) | 406 | 5837 |  |  |  |  |
|  |  | 1 | 73.4 | (70.6, 76.3) | 405 | 5836 |  |  |  |  |
|  |  | 2 | 72.0 | (69.1, 74.9) | 405 | 5836 |  |  |  |  |
|  |  | 3 | 72.7 | (69.8, 75.5) | 406 | 5836 |  |  |  |  |
|  |  | 4 | 74.0 | (71.1, 76.8) | 405 | 5835 |  |  |  |  |
|  | pooled | 0 | 70.2 | (67.3, 73.1) | 406 | 5837 |  |  |  |  |
|  |  | 1 | 73.9 | (71.1, 76.7) | 405 | 5836 |  |  |  |  |
|  |  | 2 | 73.0 | (70.2, 75.9) | 405 | 5836 |  |  |  |  |
|  |  | 3 | 73.0 | (70.1, 75.8) | 406 | 5836 |  |  |  |  |
|  |  | 4 | 74.2 | (71.4, 77.1) | 405 | 5835 |  |  |  |  |
|  | aumc | 0 | 66.9 | (64.0, 69.9) | 406 | 5837 |  |  |  |  |
|  |  | 1 | 71.0 | (68.1, 73.9) | 405 | 5836 |  |  |  |  |
|  |  | 2 | 69.9 | (67.0, 72.8) | 405 | 5836 |  |  |  |  |
|  |  | 3 | 69.8 | (66.9, 72.7) | 406 | 5836 |  |  |  |  |
|  |  | 4 | 71.4 | (68.5, 74.3) | 405 | 5835 |  |  |  |  |
| aumc | msz | 0 | 68.4 | (64.0, 72.8) | 178 | 3027 | 61.9 | (51.1, 72.8) | 30 | 1445 |
|  |  | 1 | 69.7 | (65.3, 74.1) | 178 | 3027 | 65.6 | (55.9, 75.3) | 37 | 1459 |
|  |  | 2 | 69.5 | (65.0, 73.9) | 178 | 3027 | 73.6 | (63.0, 84.3) | 28 | 1412 |
|  |  | 3 | 70.3 | (65.9, 74.7) | 179 | 3027 | 73.0 | (62.0, 83.9) | 27 | 1417 |
|  |  | 4 | 70.6 | (66.2, 75.0) | 178 | 3026 | 80.6 | (71.9, 89.3) | 36 | 1446 |
|  | olvg | 0 | 71.4 | (67.0, 75.7) | 178 | 3027 | 76.5 | (66.5, 86.5) | 30 | 1445 |
|  |  | 1 | 70.4 | (66.0, 74.8) | 178 | 3027 | 71.6 | (62.2, 81.1) | 37 | 1459 |
|  |  | 2 | 69.9 | (65.5, 74.3) | 178 | 3027 | 79.5 | (69.5, 89.5) | 28 | 1412 |
|  |  | 3 | 70.6 | (66.2, 74.9) | 179 | 3027 | 71.3 | (60.2, 82.3) | 27 | 1417 |
|  |  | 4 | 70.5 | (66.1, 74.9) | 178 | 3026 | 79.0 | (70.1, 87.9) | 36 | 1446 |
|  | pooled | 0 | 71.6 | (67.2, 75.9) | 178 | 3027 | 74.8 | (64.5, 85.0) | 30 | 1445 |
|  |  | 1 | 71.3 | (67.0, 75.7) | 178 | 3027 | 73.5 | (64.2, 82.8) | 37 | 1459 |
|  |  | 2 | 69.9 | (65.5, 74.3) | 178 | 3027 | 77.2 | (66.9, 87.5) | 28 | 1412 |
|  |  | 3 | 71.0 | (66.6, 75.3) | 179 | 3027 | 73.2 | (62.3, 84.1) | 27 | 1417 |
|  |  | 4 | 71.9 | (67.5, 76.2) | 178 | 3026 | 80.8 | (72.2, 89.5) | 36 | 1446 |
|  | aumc | 0 | 75.9 | (71.7, 80.1) | 178 | 3027 | 74.6 | (64.4, 84.9) | 30 | 1445 |
|  |  | 1 | 73.6 | (69.3, 77.8) | 178 | 3027 | 72.7 | (63.3, 82.1) | 37 | 1459 |
|  |  | 2 | 76.9 | (72.7, 81.0) | 178 | 3027 | 79.5 | (69.5, 89.5) | 28 | 1412 |
|  |  | 3 | 79.4 | (75.4, 83.3) | 179 | 3027 | 78.5 | (68.2, 88.8) | 27 | 1417 |
|  |  | 4 | 76.9 | (72.7, 81.0) | 178 | 3026 | 81.4 | (72.8, 89.9) | 36 | 1446 |

## Supplementary Table S10. Model performance per fold and their corresponding 95% confidence interval for the AUROC per fold, corresponding to the aggregated results in manuscript tables 2 and 3.

| **Dataset** | **Specialty / Model** | **MSZ** | **OLVG** | **Pooled** | **AUMC** | **Sample size** | **Incidence** |
| --- | --- | --- | --- | --- | --- | --- | --- |
| **OLVG** | cardio_surgery | 64.1 (3.1) | 71.6 (2.8) | 71.5 (2.7) | 65.9 (3.4) | 2302.6 (21.4) | 4.3 (0.2) |
|  | cardiology | 70.2 (4.0) | 71.6 (3.2) | 71.7 (3.2) | 68.3 (3.1) | 245.0 (11.9) | 10.8 (1.1) |
|  | gastroenterology | 69.1 (5.5). | 68.4 (7.4) | 68.3 (5.7) | 66.1 (7.0) | 72.5 (9.2) | 16.0 (5.2) |
|  | infrequent_sklearn | 67.7 (11.0) | 68.0 (8.7) | 70.4 (9.1) | 72.8 (7.7) | 105.2 (7.7) | 7.6 (2.1) |
|  | internal_medicine | 71.0 (3.6) | 72.8 (1.9) | 73.3 (2.7) | 69.7 (1.2) | 349.7 (13.0) | 9.7 (1.2) |
|  | neurology | 74.9 (10.0) | 73.4 (5.7) | 74.1 (7.9) | 77.1 (10.6) | 46.4 (3.9) | 9.1 (4.0) |
|  | pulmonary | 66.8 (11.6) | 67.6 (7.3) | 68.2 (8.5) | 63.2 (5.8) | 254.7 (13.0) | 8.0 (0.4) |
|  | surgery | 65.1 (1.4) | 65.9 (2.9) | 66.3 (1.7) | 64.2 (1.5) | 472.1 (15.3) | 13.0 (1.4) |
|  | unknown | - | - | - | - | 1.0 (0.0) | 33.3 (47.1) |
| **AUMC** | cardio_surgery | 70.9 (6.6) | 75.6 (3.5) | 75.9 (2.9) | 77.3 (3.2) | 1436.0 (18.1) | 2.2 (0.3) |
|  | cardiology | 67.9 (5.3) | 69.9 (5.1) | 68.3 (5.3) | 71.9 (7.5) | 192.8 (10.4) | 7.7 (0.4) |
|  | gastroenterology | 75.3 (3.9) | 64.2 (6.9) | 68.7 (8.0) | 70.6 (9.8) | 33.6 (3.0) | 8.4 (5.6) |
|  | infrequent_sklearn | 66.1 (1.0) | 62.5 (1.6) | 64.7 (2.2) | 67.4 (3.1) | 440.0 (14.1) | 8.4 (0.6) |
|  | intensive_care | 68.7 (1.7) | 72.0 (2.9) | 70.2 (3.1) | 72.5 (5.6) | 127.2 (10.0) | 10.3 (1.8) |
|  | internal_medicine | 70.8 (3.2) | 71.0 (3.1) | 71.6 (3.4) | 71.0 (4.1) | 230.6 (6.7) | 8.8 (2.3) |
|  | neurology | 66.5 (7.7) | 64.4 (9.6) | 64.5 (8.7) | 66.7 (9.0) | 109.2 (9.4) | 8.7 (2.3) |
|  | pulmonary | 66.8 (8.0) | 66.4 (6.1) | 66.2 (8.5) | 68.8 (5.4) | 128.0 (7.1) | 10.3 (1.7) |
|  | surgery | 63.9 (2.2) | 62.7 (3.2) | 63.7 (1.8) | 64.6 (3.5) | 333.8 (16.0) | 10.9 (0.9) |

## Supplementary Table 11. Performance by AUROC of each hospital’s prediction model stratified for specialty of admission. Results from the MSZ and Pooled datasets are removed because they are identical to the overall MSZ and stratified OLVG results because of the unavailability of the specialty of admission in MSZ.

| **Dataset/Model** | **MSZ** | **OLVG** | **Pooled** | **AUMC** | **Sample size** | **Incidence** |
| --- | --- | --- | --- | --- | --- | --- |
| **MSZ** | 70.9 (1.4) | 70.3 (1.9) | 70.8 (1.4) | 68.9 (2.3) | 1991.8 (0.4) | 7.1 (0.0) |
| **OLVG** | 68.2 (3.1) | 70.2 (3.0) | 70.1 (2.8) | 66.8 (2.3) | 1541.8 (0.4) | 10.8 (0.0) |
| **Pooled** | 69.4 (1.2) | 70.4 (1.6) | 71.3 (1.2) | 68.1 (1.6) | 3533.6 (0.8) | 8.7 (0.0) |
| **AUMC** | 66.7 (1.8) | 66.5 (1.9) | 66.8 (1.6) | 68.0 (2.1) | 1591.0 (0.0) | 9.2 (0.0) |

## Supplementary Table 12. Model performance by AUROC score of local and pooled models on each dataset, when training and testing on datasets without cardiac surgery patients. Reported values are weighted means with standard deviation, averaged over 5 folds.

| **Dataset/Model** | **MSZ** | **OLVG** | **Pooled** | **AUMC** | **Sample size** | **Incidence** |
| --- | --- | --- | --- | --- | --- | --- |
| **MSZ** | 70.9 (1.6) | 70.6 (1.9) | 70.9 (1.8) | 69.2 (1.5) | 1991.8 (0.4) | 7.1 (0.0) |
| **OLVG** | 68.5 (3.9) | 69.5 (2.5) | 69.9 (2.7) | 66.4 (2.5) | 1541.8 (0.4) | 10.8 (0.0) |
| **Pooled** | 69.6 (1.9) | 70.1 (1.5) | 71.0 (1.5) | 68.1 (1.4) | 3533.6 (0.8) | 8.7 (0.0) |
| **AUMC** | 67.0 (1.0) | 66.1 (1.5) | 66.6 (1.1) | 68.1 (1.5) | 1591.0 (0.0) | 9.2 (0.0) |

## Supplementary Table 13. Performance by AUROC score of each hospital’s prediction model on each dataset, including cardiac surgery patients in the training set and excluding cardiac surgery patients in the testing set.

| **Dataset** | **Hospital x Hospital** | **MSZ** | **OLVG** | **Pooled** |
| --- | --- | --- | --- | --- |
| **MSZ** | **OLVG** | 0.730 |  |  |
|  | **Pooled** | 0.894 | 0.932 |  |
|  | **AUMC** | 0.867 | 0.804 | 0.896 |
| **OLVG** | **OLVG** | **0.481** |  |  |
|  | **Pooled** | 0.627 | 0.964 |  |
|  | **AUMC** | 0.891 | 0.646 | 0.765 |
| **Pooled** | **OLVG** | **0.436** |  |  |
|  | **Pooled** | 0.632 | 0.935 |  |
|  | **AUMC** | 0.863 | 0.639 | 0.788 |
| **AUMC** | **OLVG** | **0.771** |  |  |
|  | **Pooled** | 0.872 | 0.970 |  |
|  | **AUMC** | **0.705** | 0.746 | 0.763 |

## Supplementary Table S14. Collinearity between out-of-distribution models on local and pooled datasets.

| **Dataset** | **Hospital x Hospital** | **MSZ** | **OLVG** | **Pooled** |
| --- | --- | --- | --- | --- |
| **MSZ** | **OLVG** | 0.821 |  |  |
|  | **Pooled** | 0.956 | 0.942 |  |
|  | **AUMC** | 0.834 | 0.793 | 0.859 |
| **OLVG** | **OLVG** | 0.874 |  |  |
|  | **Pooled** | 0.961 | 0.968 |  |
|  | **AUMC** | 0.906 | 0.853 | 0.906 |
| **Pooled** | **OLVG** | 0.818 |  |  |
|  | **Pooled** | 0.954 | 0.943 |  |
|  | **AUMC** | 0.876 | 0.816 | 0.890 |
| **AUMC** | **OLVG** | 0.867 |  |  |
|  | **Pooled** | 0.955 | 0.970 |  |
|  | **AUMC** | **0.803** | 0.716 | 0.775 |

## Supplementary Table 15. Collinearity between out-of-distribution models on local and pooled datasets when excluding cardiac surgery patients.

| **Group/Model** | **MSZ** | **OLVG** | **Pooled** | **AUMC** | **Sample size** | **Incidence** |
| --- | --- | --- | --- | --- | --- | --- |
| **5.1** | 61.6 (5.5) | 65.6 (7.3) | 65.1 (7.4) | 79.3 (3.6) | 1010.0 (31.1) | 3.0 (0.6) |
| **6.3** | 62.0 (3.6) | 63.6 (2.2) | 64.2 (2.3) | 73.4 (4.2) | 1010.0 (33.9) | 4.7 (0.7) |
| **8.5** | 66.8 (1.3) | 66.3 (1.5) | 67.1 (1.2) | 70.1 (2.4) | 1009.7 (27.1) | 10.0 (0.5) |

## Supplementary Table 16. Model performance by AUROC score on AUMC dataset, stratified over three groups of out-of-distribution scores according to the Pooled model. Reported values are weighted means with standard deviation, averaged over 5 folds. The group labels represent median out-of-distribution scores for each group.

| **OOD detector** | **Filter** | **MSZ** | **OLVG** | **Pooled** | **AUMC** | **Sample size** | **Incidence** |
| --- | --- | --- | --- | --- | --- | --- | --- |
| **MSZ** | **in-domain** | 69.6 (1.0) | 70.5 (0.4) | 71.1 (0.5) | 76.6 (2.1) | 2967.4 (8.6) | 5.7 (0.0) |
|  | **out-domain** | 58.7 (7.8) | 57.1 (9.5) | 58.6 (9.4) | 61.3 (7.6) | 60.7 (8.8) | 13.5 (1.7) |
| **OLVG** | **in-domain** | 69.3 (0.8) | 70.2 (0.4) | 70.8 (0.6) | 76.5 (2.0) | 2937.2 (8.8) | 5.7 (0.1) |
|  | **out-domain** | 67.7 (4.3) | 67.7 (3.5) | 69.1 (3.3) | 67.6 (5.8) | 90.5 (8.9) | 13.2 (2.0) |
| **Pooled** | **in-domain** | 69.4 (0.9) | 70.3 (0.5) | 70.9 (0.6) | 76.5 (2.2) | 2953.2 (8.0) | 5.7 (0.1) |
|  | **out-domain** | 66.2 (4.5) | 64.4 (3.7) | 66.2 (5.1) | 66.4 (5.1) | 74.5 (8.1) | 13.6 (2.5) |
| **AUMC** | **in-domain** | 69.4 (0.8) | 70.3 (0.3) | 70.9 (0.5) | 76.4 (2.0) | 2996.4 (6.6) | 5.8 (0.1) |
|  | **out-domain** | 66.7 (4.4) | 61.8 (6.7) | 61.8 (7.1) | 67.1 (8.6) | 31.8 (5.8) | 16.4 (7.1) |

## Supplementary Table 17. Performance on the AUMC dataset for each hospital’s prediction model, stratified for out of domain score of each hospital.

| **Median OOD score:** |  | **5.11** | | **6.3** | | **8.54** | |
| --- | --- | --- | --- | --- | --- | --- | --- |
| **Feature** | **Description** | **Missing, N (%)** | **value** | **Missing, N (%)** | **value** | **Missing, N (%)** | **value** |
| **Number of records** | **N (%)** | - | 5045 (100.0%) | - | 5044 (100.0%) | - | 5045 (100.0%) |
| **ABP diastolic, last 24h, mean** | **Median [IQR]** | 455.0 (9.02%) | 61.19 [56.36, 66.62] | 227.0 (4.5%) | 61.69 [56.08, 68.73] | 360.0 (7.14%) | 62.19 [55.92, 70.24] |
| **ABP systolic, last 24h, mean** | **Median [IQR]** | 455.0 (9.02%) | 125.31 [114.37, 135.25] | 227.0 (4.5%) | 126.19 [114.06, 139.5] | 361.0 (7.16%) | 127.17 [113.94, 142.22] |
| **ALAT, last 24h, mean** | **Median [IQR]** | 4028.0 (79.84%) | 26.0 [17.5, 43.0] | 3608.0 (71.53%) | 25.33 [17.0, 46.0] | 2901.0 (57.5%) | 36.58 [19.88, 82.0] |
| **APTT, last 24h, mean** | **Median [IQR]** | 705.0 (13.97%) | 36.25 [33.33, 39.33] | 602.0 (11.93%) | 38.0 [34.0, 43.0] | 793.0 (15.72%) | 41.0 [35.0, 53.33] |
| **ASAT, last 24h, mean** | **Median [IQR]** | 4057.0 (80.42%) | 40.0 [27.0, 59.0] | 3646.0 (72.28%) | 38.0 [24.0, 61.75] | 2943.0 (58.33%) | 42.17 [25.0, 80.0] |
| **Age** | **Median [IQR]** | 0.0 (0.0%) | 66.89 [58.37, 74.0] | 0.0 (0.0%) | 67.0 [55.75, 75.51] | 0.0 (0.0%) | 65.9 [52.53, 75.0] |
| **Albumin, last 24h, mean** | **Median [IQR]** | 646.0 (12.8%) | 26.0 [23.0, 28.0] | 301.0 (5.97%) | 24.0 [21.0, 28.0] | 234.0 (4.64%) | 23.0 [19.0, 27.0] |
| **Alkaline Phosphatase, last 24h, mean** | **Median [IQR]** | 4237.0 (83.98%) | 59.5 [48.0, 74.62] | 3844.0 (76.21%) | 63.0 [48.88, 86.0] | 3169.0 (62.81%) | 95.0 [63.0, 162.0] |
| **BE, last 24h, mean** | **Median [IQR]** | 437.0 (8.66%) | 0.96 [-0.76, 1.9] | 178.0 (3.53%) | 1.22 [-1.22, 2.73] | 153.0 (3.03%) | 2.03 [0.14, 4.18] |
| **BMI, last 24h, mean** | **Median [IQR]** | 2282.0 (45.23%) | 26.15 [24.3, 28.38] | 2380.0 (47.18%) | 25.71 [23.39, 28.58] | 2935.0 (58.18%) | 25.12 [22.66, 28.05] |
| **Bilirubin, total, last 24h, mean** | **Median [IQR]** | 3330.0 (66.01%) | 8.0 [6.0, 11.0] | 2898.0 (57.45%) | 8.0 [6.0, 12.0] | 2354.0 (46.66%) | 8.0 [5.5, 13.0] |
| **CK-MB mass, last 24h, is measured** | **N (%)** | 0.0 (0.0%) | 3516 (69.69%) | 0.0 (0.0%) | 2801 (55.53%) | 0.0 (0.0%) | 1693 (33.56%) |
| **CK-MB, last overall** | **Median [IQR]** | 970.0 (19.23%) | 15.0 [8.9, 23.9] | 1232.0 (24.43%) | 14.8 [7.3, 25.12] | 1762.0 (34.93%) | 10.9 [4.5, 24.25] |
| **CRP, last 24h, mean** | **Median [IQR]** | 3010.0 (59.66%) | 34.0 [17.0, 62.42] | 2623.0 (52.0%) | 46.33 [19.0, 103.0] | 2053.0 (40.69%) | 65.0 [26.0, 139.0] |
| **Calcium, ionized, last 24h, mean** | **Median [IQR]** | 1422.0 (28.19%) | 1.17 [1.14, 1.2] | 1020.0 (20.22%) | 1.17 [1.13, 1.21] | 902.0 (17.88%) | 1.18 [1.13, 1.22] |
| **Cardiac Output, last 24h, is measured** | **N (%)** | 0.0 (0.0%) | 2250 (44.6%) | 0.0 (0.0%) | 1796 (35.61%) | 0.0 (0.0%) | 900 (17.84%) |
| **Cardiac Output, last 24h, max** | **Median [IQR]** | 2795.0 (55.4%) | 6.3 [5.39, 7.3] | 3248.0 (64.39%) | 5.8 [4.9, 6.79] | 4145.0 (82.16%) | 5.4 [4.52, 6.5] |
| **Cardiac Output, last 24h, min** | **Median [IQR]** | 2795.0 (55.4%) | 4.8 [4.1, 5.6] | 3248.0 (64.39%) | 4.3 [3.6, 5.1] | 4145.0 (82.16%) | 3.9 [3.2, 4.8] |
| **Chloride** | **Median [IQR]** | 1383.0 (27.41%) | 107.14 [105.0, 109.12] | 948.0 (18.79%) | 107.65 [104.62, 110.5] | 781.0 (15.48%) | 106.25 [102.25, 110.14] |
| **Creatinine, last overall** | **Median [IQR]** | 206.0 (4.08%) | 76.0 [63.0, 91.0] | 39.0 (0.77%) | 77.0 [60.0, 97.0] | 33.0 (0.65%) | 79.0 [58.0, 117.0] |
| **FiO2, last 24h, mean** | **Median [IQR]** | 1765.0 (34.99%) | 40.29 [39.95, 42.86] | 2000.0 (39.65%) | 40.51 [40.0, 45.45] | 2790.0 (55.3%) | 41.46 [39.98, 49.61] |
| **GCS, last 24h, max** | **Median [IQR]** | 1426.0 (28.27%) | 15.0 [15.0, 15.0] | 1587.0 (31.46%) | 15.0 [15.0, 15.0] | 1662.0 (32.94%) | 15.0 [15.0, 15.0] |
| **GCS, last 24h, min** | **Median [IQR]** | 1426.0 (28.27%) | 15.0 [14.0, 15.0] | 1587.0 (31.46%) | 15.0 [14.0, 15.0] | 1662.0 (32.94%) | 15.0 [13.0, 15.0] |
| **Glucose, last 24h, min** | **Median [IQR]** | 357.0 (7.08%) | 6.8 [5.9, 7.8] | 114.0 (2.26%) | 6.3 [5.4, 7.4] | 85.0 (1.68%) | 6.1 [5.2, 7.0] |
| **HCO3-, arterial, last 24h, mean** | **Median [IQR]** | 635.0 (12.59%) | 23.38 [22.32, 24.6] | 413.0 (8.19%) | 23.3 [21.8, 25.24] | 460.0 (9.12%) | 24.0 [21.56, 27.3] |
| **Hb, last 24h, mean** | **Median [IQR]** | 195.0 (3.87%) | 6.91 [6.34, 7.58] | 29.0 (0.57%) | 6.72 [6.12, 7.5] | 21.0 (0.42%) | 6.49 [5.85, 7.3] |
| **Heart rate, last 24h, mean** | **Median [IQR]** | 99.0 (1.96%) | 78.33 [71.16, 85.45] | 6.0 (0.12%) | 80.0 [71.27, 89.48] | 3.0 (0.06%) | 84.63 [74.5, 95.55] |
| **LDH, last 24h, mean** | **Median [IQR]** | 4092.0 (81.11%) | 244.0 [190.5, 307.5] | 3743.0 (74.21%) | 248.0 [188.0, 350.0] | 3126.0 (61.96%) | 291.0 [206.0, 418.0] |
| **Lactate, last overall** | **Median [IQR]** | 2338.0 (46.34%) | 1.2 [0.9, 1.7] | 1719.0 (34.08%) | 1.2 [0.9, 1.79] | 902.0 (17.88%) | 1.2 [0.9, 1.7] |
| **Length of Stay** | **Median [IQR]** | 0.0 (0.0%) | 22.9 [20.23, 29.42] | 0.0 (0.0%) | 24.3 [20.98, 52.0] | 0.0 (0.0%) | 47.47 [23.0, 154.47] |
| **Leukocytes, last 24h, mean** | **Median [IQR]** | 216.0 (4.28%) | 12.9 [10.45, 15.55] | 44.0 (0.87%) | 12.5 [9.7, 15.57] | 44.0 (0.87%) | 12.15 [9.14, 16.0] |
| **Magnesium,last 24h, mean** | **Median [IQR]** | 647.0 (12.82%) | 0.97 [0.84, 1.09] | 544.0 (10.79%) | 0.92 [0.79, 1.09] | 809.0 (16.04%) | 0.86 [0.76, 1.02] |
| **O2 flow, last 24h, mean** | **Median [IQR]** | 406.0 (8.05%) | 4.0 [2.92, 5.0] | 374.0 (7.41%) | 4.62 [3.0, 5.25] | 521.0 (10.33%) | 4.71 [3.0, 6.0] |
| **O2 saturation, last 24h, mean** | **Median [IQR]** | 307.0 (6.09%) | 97.0 [95.5, 98.14] | 144.0 (2.85%) | 96.75 [94.75, 98.1] | 120.0 (2.38%) | 96.3 [93.8, 97.8] |
| **PEEP, last 24h, max** | **Median [IQR]** | 965.0 (19.13%) | 5.7 [5.2, 8.0] | 729.0 (14.45%) | 6.1 [5.2, 8.4] | 893.0 (17.7%) | 8.3 [5.4, 12.0] |
| **PaCO2, last 24h, mean** | **Median [IQR]** | 608.0 (12.05%) | 39.0 [36.83, 41.5] | 390.0 (7.73%) | 39.0 [36.0, 42.0] | 436.0 (8.64%) | 38.66 [35.0, 43.0] |
| **PaO2/FiO2 ratio, last overall** | **Median [IQR]** | 792.0 (15.7%) | 277.5 [222.5, 340.0] | 550.0 (10.9%) | 272.75 [207.5, 357.14] | 576.0 (11.42%) | 250.0 [190.0, 350.0] |
| **Phosphate, last 24h, mean** | **Median [IQR]** | 664.0 (13.16%) | 0.99 [0.83, 1.14] | 413.0 (8.19%) | 0.98 [0.8, 1.17] | 404.0 (8.01%) | 1.07 [0.86, 1.3] |
| **Potassium, last 24h, mean** | **Median [IQR]** | 346.0 (6.86%) | 4.16 [3.97, 4.37] | 102.0 (2.02%) | 4.1 [3.88, 4.38] | 82.0 (1.63%) | 4.1 [3.86, 4.38] |
| **RASS, last 24h, max** | **Median [IQR]** | 4030.0 (79.88%) | 0.0 [0.0, 0.0] | 3889.0 (77.1%) | 0.0 [0.0, 0.0] | 3599.0 (71.34%) | 0.0 [0.0, 0.0] |
| **RASS, last 24h, min** | **Median [IQR]** | 4030.0 (79.88%) | 0.0 [-1.0, 0.0] | 3889.0 (77.1%) | 0.0 [-1.0, 0.0] | 3599.0 (71.34%) | 0.0 [-1.0, 0.0] |
| **Respiratory rate, last 24h, mean** | **Median [IQR]** | 613.0 (12.15%) | 15.69 [14.2, 17.81] | 442.0 (8.76%) | 16.19 [14.36, 18.89] | 527.0 (10.45%) | 18.24 [15.56, 21.92] |
| **Sex, Male** | **N (%)** | 0.0 (0.0%) | 3836 (76.04%) | 0.0 (0.0%) | 3218 (63.8%) | 0.0 (0.0%) | 3057 (60.59%) |
| **Sodium, last 24h, mean** | **Median [IQR]** | 346.0 (6.86%) | 138.43 [136.83, 140.16] | 106.0 (2.1%) | 138.82 [136.67, 141.0] | 85.0 (1.68%) | 138.67 [136.0, 141.48] |
| **TV/kg, last 24h, mean** | **Median [IQR]** | 1098.0 (21.76%) | 7.18 [6.55, 7.9] | 912.0 (18.08%) | 7.21 [6.46, 8.11] | 1061.0 (21.03%) | 7.13 [6.33, 8.11] |
| **Thrombocytes, last 24h, mean** | **Median [IQR]** | 391.0 (7.75%) | 172.0 [140.0, 214.0] | 192.0 (3.81%) | 172.0 [129.0, 230.5] | 176.0 (3.49%) | 203.0 [137.5, 313.0] |
| **Tracheobronchial toilet, last 24, count** | **Median [IQR]** | 4617.0 (91.52%) | 1.0 [1.0, 2.0] | 4301.0 (85.27%) | 2.0 [1.0, 3.0] | 4012.0 (79.52%) | 3.0 [2.0, 6.0] |
| **Ureum, last 24h, mean** | **Median [IQR]** | 2779.0 (55.08%) | 6.1 [4.9, 7.6] | 2317.0 (45.94%) | 6.2 [4.4, 8.7] | 1786.0 (35.4%) | 8.1 [5.25, 13.1] |
| **Ureum/Creatinine ratio, last 24h, mean** | **Median [IQR]** | 2786.0 (55.22%) | 0.08 [0.07, 0.1] | 2327.0 (46.13%) | 0.08 [0.06, 0.11] | 1801.0 (35.7%) | 0.09 [0.06, 0.13] |
| **Urine production, last 24h, total** | **Median [IQR]** | 128.0 (2.54%) | 1.67 [1.25, 2.23] | 34.0 (0.67%) | 1.69 [1.21, 2.36] | 110.0 (2.18%) | 1.71 [1.12, 2.55] |
| **age_cat_50-70** | **N (%)** |  | 2575 (51.04%) |  | 2065 (40.94%) |  | 1970 (39.05%) |
| **age_cat_70-80** | **N (%)** |  | 1583 (31.38%) |  | 1498 (29.7%) |  | 1373 (27.22%) |
| **age_cat_<50** | **N (%)** |  | 536 (10.62%) |  | 920 (18.24%) |  | 1121 (22.22%) |
| **age_cat_>80** | **N (%)** |  | 351 (6.96%) |  | 561 (11.12%) |  | 581 (11.52%) |
| **gGT, last 24h, mean** | **Median [IQR]** | 4191.0 (83.07%) | 26.0 [17.0, 46.0] | 3774.0 (74.82%) | 32.25 [17.0, 68.0] | 3088.0 (61.21%) | 68.0 [28.0, 163.0] |
| **icu_specialty_anesthesiology** | **N (%)** |  |  |  | 1 (0.02%) |  | 1 (0.02%) |
| **icu_specialty_cardio_surgery** | **N (%)** |  | 3315 (65.71%) |  | 2435 (48.28%) |  | 1429 (28.33%) |
| **icu_specialty_cardiology** | **N (%)** |  | 188 (3.73%) |  | 311 (6.17%) |  | 462 (9.16%) |
| **icu_specialty_ent** | **N (%)** |  | 74 (1.47%) |  | 85 (1.69%) |  | 149 (2.95%) |
| **icu_specialty_gastroenterology** | **N (%)** |  | 43 (0.85%) |  | 44 (0.87%) |  | 80 (1.59%) |
| **icu_specialty_gynecology** | **N (%)** |  | 8 (0.16%) |  | 39 (0.77%) |  | 87 (1.72%) |
| **icu_specialty_intensive_care** | **N (%)** |  | 117 (2.32%) |  | 193 (3.83%) |  | 322 (6.38%) |
| **icu_specialty_internal_medicine** | **N (%)** |  | 161 (3.19%) |  | 317 (6.28%) |  | 674 (13.36%) |
| **icu_specialty_mka** | **N (%)** |  | 5 (0.1%) |  | 9 (0.18%) |  | 14 (0.28%) |
| **icu_specialty_neuro_surgery** | **N (%)** |  | 139 (2.76%) |  | 224 (4.44%) |  | 194 (3.85%) |
| **icu_specialty_neurology** | **N (%)** |  | 132 (2.62%) |  | 224 (4.44%) |  | 186 (3.69%) |
| **icu_specialty_oncology** | **N (%)** |  | 4 (0.08%) |  | 7 (0.14%) |  | 21 (0.42%) |
| **icu_specialty_ophtalmology** | **N (%)** |  |  |  | 3 (0.06%) |  | 2 (0.04%) |
| **icu_specialty_orthopedic_surgery** | **N (%)** |  | 11 (0.22%) |  | 59 (1.17%) |  | 36 (0.71%) |
| **icu_specialty_other** | **N (%)** |  | 10 (0.2%) |  | 7 (0.14%) |  | 10 (0.2%) |
| **icu_specialty_plastic_surgery** | **N (%)** |  | 4 (0.08%) |  | 7 (0.14%) |  | 11 (0.22%) |
| **icu_specialty_psychiatry** | **N (%)** |  |  |  | 3 (0.06%) |  |  |
| **icu_specialty_pulmonary** | **N (%)** |  | 110 (2.18%) |  | 165 (3.27%) |  | 363 (7.2%) |
| **icu_specialty_rehabilitation_medicine** | **N (%)** |  |  |  | 1 (0.02%) |  |  |
| **icu_specialty_rheumatology** | **N (%)** |  |  |  | 2 (0.04%) |  | 1 (0.02%) |
| **icu_specialty_surgery** | **N (%)** |  | 487 (9.65%) |  | 552 (10.94%) |  | 626 (12.41%) |
| **icu_specialty_trauma_surgery** | **N (%)** |  | 91 (1.8%) |  | 151 (2.99%) |  | 144 (2.85%) |
| **icu_specialty_urology** | **N (%)** |  | 20 (0.4%) |  | 40 (0.79%) |  | 53 (1.05%) |
| **icu_specialty_vascular_surgery** | **N (%)** |  | 126 (2.5%) |  | 165 (3.27%) |  | 180 (3.57%) |
| **Length of Stay, 1-3 days** | **N (%)** |  | 1462 (28.98%) |  | 1731 (34.32%) |  | 1553 (30.78%) |
| **Length of Stay, 12-24 hours** | **N (%)** |  | 3114 (61.72%) |  | 2403 (47.64%) |  | 1467 (29.08%) |
| **Length of Stay, 3-7 days** | **N (%)** |  | 386 (7.65%) |  | 632 (12.53%) |  | 870 (17.24%) |
| **Length of Stay, 4-12 hours** | **N (%)** |  |  |  | 5 (0.1%) |  |  |
| **Length of Stay, 7-14 days** | **N (%)** |  | 76 (1.51%) |  | 230 (4.56%) |  | 641 (12.71%) |
| **Length of Stay, >14 days** | **N (%)** |  | 7 (0.14%) |  | 43 (0.85%) |  | 514 (10.19%) |
| **pH arterial, last overall** | **Median [IQR]** | 458.0 (9.08%) | 7.42 [7.4, 7.44] | 231.0 (4.58%) | 7.42 [7.38, 7.45] | 165.0 (3.27%) | 7.43 [7.39, 7.46] |

## Supplementary Table 18. Patient characteristics of AUMC patients within three bins of out-of-distribution score based on the Pooled model.
